# Supplementary material for: Experimental demonstration of tunable hybrid improper ferroelectricity in double-perovskite superlattice films
Source: Nat Commun. 2024 Jul 2;15:5549. doi: 10.1038/s41467-024-49707-x (PMC11219787; doi:10.1038/s41467-024-49707-x)
Supplement: Supplementary file 1 — Supplementary Information [file 41467_2024_49707_MOESM1_ESM.pdf]

Supplementary Information for

Experimental demonstration of tunable hybrid improper ferroelectricity in double-perovskite superlattice films

Yaoxiang Jiang[1,†], Jianguo Niu[1,†], Cong Wang[2,†,*], Donglai Xue[1], Xiaohui Shi[1], Weibo Gao[3,*], Shifeng Zhao[1,*]

[1]Inner Mongolia Key Lab of Nanoscience and Nanotechnology, Inner Mongolia University, Hohhot 010021, PR China;

[2]College of Mathematics and Physics, Beijing University of Chemical Technology, Beijing 100029, China;

[3]Division of Physics and Applied Physics, School of Physical and Mathematical Sciences, Nanyang Technological University, Singapore 637371, Singapore

[†]These authors contributed equally: Yaoxiang Jiang, Jianguo Niu, Cong Wang
[*]Corresponding author Email:
wangcongphysics@mail.buct.edu.cn; wbgao@ntu.edu.sg; zhsf@imu.edu.cn

Contents

Supplementary Note 1: Superlattices growth and structural characterizations..........................1
Supplementary Note 2: Ferroelectricity characterizations of the superlattice films with different thickness...13
Supplementary Note 3: Estimation of piezoelectric coefficient (d_{33})......................................23
Supplementary Note 4: X-ray photoelectron spectroscopy and Raman spectra.......................26
Supplementary Note 5: DFT simulations for octahedral distortion..29
Supplementary Appendix 1: Landau-Ginsburg-Devonshire theory for ferroelectric phase transition...34
Supplementary References..36

Supplementary Notes and Figures

Supplementary Note 1: Superlattices growth and structural characterizations.

The double-perovskite La_2NiMnO_6/La_2CoMnO_6 superlattice films were grown on the etched substrates with the TiO_2-terminated and terraced surface. Supplementary Figure S1a shows sixteen periods of RHEED intensity, starting from deposition, collected from principal diffraction of reflex peak (00). All the oscillations of RHEED intensity are quite sharp, as shown in the inset, indicating the layer-by-layer growth mode for epitaxial growth. The growth of LNMO and LCMO layers can be easily identified from the trend and amplitude of the intensity oscillations because of the distinct local thermodynamic equilibrium conditions for different chemical elements[1]. The terraced surface of superlattice films indicates the highly ordered growth at atomic scale for double perovskites (Supplementary Figure S1b). The additional diffraction fringes indexed as half-integer plane (k/2, k/2) are the two-fold superstructure streaks from a $2^{1/2}a\times2^{1/2}a$ superstructure (Supplementary Figure S1c). The corresponding peaks in intensity profile are much clearer, and these streaks indicate a rock-salt double-perovskite structure with high ordering of B-site cations[2].

There are three types of epitaxial modes for double perovskites (LNMO and LCMO) to grow on Nb:STO substrates, according to the extra diffraction spots in SAED, which is the same as discussed in FFT image. It can be marked as type-I, -II and -III, i.e., the growth modes of $[001](001)_{SL}//[001](001)_{Sub}$, $[001](110)_{SL}//[010](001)_{Sub}$ and $[001](110)_{SL}//[100](001)_{Sub}$, respectively (Supplementary Figure S2). Notably, the additional diffraction spots of type-II and -III are more obvious than that of type-I, which indicates a relatively few proportions of type-I epitaxial structures within the tested region. These extra diffraction spots originate from the superstructures of B-site ordered double perovskites. In fact, the extinction law of space group $P2_1/n$ is (h 0 l) and (0 k 0), where $h + l$ = odd number; k = odd number, respectively. For the type-I and -II, the simplest extra diffraction spot originates from (00l), where l is odd number, because (001) is the close-packed plane for ordered B-site cations except the extinction of primary diffraction. While for the type-III, the emitted electron beam is along [001] zone axis for generating diffraction, the second close-packed plane is (0 k 0). Thus, the extra diffraction spot originates from (0 k 0), where k is odd number. Furthermore, EDS mappings were tested to indicate the good uniformity and

stoichiometry of *B*-site cations in double perovskites. As shown in Supplementary Figure S3, the elemental distribution is homogeneous and the ratio of *B*-site atoms close to 2:1:1. These results contribute to generate extra diffraction spots from the close-packed plane with *B*-site orderings in superlattices.

The corresponding lattice mismatch of three epitaxial modes is calculated by the formula of $f = [(d_f - d_s)/d_f]\times 100\%$, where d_f and d_s are the lattice parameters of the thin films and substrates. As shown in the schematic, when the superlattice films are grown in a combination of various epitaxial modes, the epitaxial strain comes from the lattice mismatch between not only the films and the substrate, but also the superlattices with different orientations in the films (Supplementary Figure S4). Therefore, the large strain transfer distances can be realized in such multiple epitaxial patterns even with small lattice mismatch. As expected, the structural design and strain control provide a new dimension for achieving long-range modulation of the octahedral rotation and tilting.

The azimuthal Φ-scan around the $(111)_{pc}$ reflection of the superlattice films with different thicknesses shows the uniform in-plane crystallinity and fourfold rotational symmetry (Supplementary Figure S5a). All rocking curves detected from the $(002)_{pc}$ reflection show the single peak without the feedback of superlattice periods and impurity phases, which implies no clear heterogeneous interfaces in the superlattice films (Supplementary Figure S5b). This result further indicates that the multiple epitaxial growth with mixed crystalline orientations can generate the homogeneous structures in thin films due to the unique lattice parameters of LNMO and LCMO (i.e., $2^{1/2}a \approx 2^{1/2}b \approx c$). The average value of full width at half maximum (FWHM) is approximately 0.06°, indicating a high crystalline quality out of plane. This excellent crystallinity is closely correlated to the high degree of *B*-site ordering in DPs during the ozone-assisted growth process. However, the FWHM values gradually decreases with the film thickness increasing, which means the decreasing OP crystallinity and reduced epitaxy in thick films. Therefore, the strain relaxation in thick films is correlated to the OP crystallinity since the mismatch strain highly depends on good film epitaxy.

We utilized an ozone-assisted growth method to achieve the growth of high-quality double-perovskite superlattices. As shown in Supplementary Figure S6, the local HAADF images reveal fully epitaxial structures with the coherent growth for the LNMO and LCMO superlattice layers. Moreover, for the local IP and OP displacement fields (u_{xx} and u_{yy}) of atomic columns, the displacement distributions of A-site cations are almost unchanged along the IP and OP directions, while changing slightly for B-site cations. This difference in the atomic projection, to some extent, indicates the titling/rotation of BO_6 octahedron in the superlattice films, which can usually affect the ferroic order parameters in the system. The reliable rotation or tilting of the oxygen octahedron is analyzed by the changes in B-O-B bond angles, according to the ABF STEM images for oxygen distribution (see Figure 2h). Supplementary Figure S7 shows the strain maps processed in grayscale to reflect the strain distribution clearly. According to the practical in-plane and out-of-plane crystal axes, the appropriate cutting lines were employed to obtain the intensity profiles of strain.

Moreover, we have tracked the strain within the superlattice films to fully explore the strain relaxation in different samples. Supplementary Figure S8 shows the local geometric phase analysis and the corresponding lattice dislocations of the SL_{60} films. The TEM images with irregular lattice registries imply the local disordering of the superlattices, and the reconstructed lattice images are more visible. Even though the quality of double-perovskite growth was improved by ozone-assisted methods, the local lattice disorders could not be completely avoided, which fully illustrates the difficulty of preparing double-perovskite superlattices. Two non-colinear reciprocal space vectors ($\mathbf{g}_1$ and $\mathbf{g}_2$) with large intensities of power spectra were selected to reach an excellent signal-to-noise ratio for GPA. According to the strain components of ε_{xx} and ε_{yy} in Supplementary Figure S8c, d, the strain only accumulates in the disordered regions of the films. We then analyze the monochromatic filtered IFFT images of IP and OP planes. Obvious T-type dislocations are observed in the strain regions. Moreover, the continuous but curved lattice fringes indicate some degree of lattice distortion that does not form a typical defect type in the films. Therefore, the

macroscopic strain is released through the formation of T-type dislocation and lattice deformation with bending fringes. These results hinder the long-range propagation of epitaxial strains caused by the lattice mismatch, thereby leading to the local strain fluctuations. In order to further reveal the physical process of epitaxial strain release with increasing film thickness, GPA was performed on a larger lattice region of the SL_{90} (Supplementary Figure S9). The calculated GPA displays a standard geometric correlation between strain field (ε_{xx} and ε_{yy}), phase contrast ($\boldsymbol{P}g_1$ and $\boldsymbol{P}g_2$) and reciprocal lattice vectors ($\mathbf{g}_1$ and $\mathbf{g}_2$). The FFT of three different regions clearly demonstrates the changing process of the lattice parameters. Epitaxial strain is released through the dislocations and lattice distortions along the boundaries of different regions. Notably, the phase contrast is significantly intermittent, indicating the influence caused by the lattice defects in the superlattice films. Interestingly, the dislocations and lattice distortions occur only in the out-of-plane direction, according to the monochromatic filtered IFFT images of IP and OP planes. Thus, this result illustrates that strain release is primarily accomplished by the lattice defects and driven by the increasing film thickness, which is closely related to the decrease in the epitaxy of thick films.

Supplementary Figure S10 shows annular bright-field (ABF) STEM images of the SL_{60} films along different crystal axis directions of SL-[110] and -[0 -1 1]. According to the corresponding schematic of oxygen distribution, the layered structure of oxygen columns measured along SL-[110] axis cannot be observed clearly. The stacking of *A*- and *B*-site atoms in close-packed plane does not provide the sufficient atomic gaps for the oxygen atoms with lighter masses to reflect the contrast of the sublattice projections. Consequently, despite the fact that the octahedron is in a simple geometric perspective, the tilting or rotation of the oxygen octahedron cannot be determined. While, along SL-[0 -1 1] axis, the elongated and misaligned oxygen sublattices are arranged in a zigzag-like pattern. This result significantly indicates the qualitative octahedral distortions in the superlattice films. The layered structure of oxygen columns forms a close-packed plane which is highlighted both in the ABF

image and structural schematic by the red dashed line and blue rectangular box, respectively. However, the quantitative analysis of BO_6 octahedral distortions cannot be proceeded because the geometrical position of octahedron is too complex to measure any bond angles of *B*-O-*B*' for OOR/OOT.

The best visualization of recording ABF-STEM image to analyze OOR is along the STO-[110] axis, i.e., SL-[100] or [010] axes. Supplementary Figure S11 shows the illustrations of the geometric structure for the sample preparation by using focused ion beam (FIB) and the STO-[110] axis for the ABF measurements. The films are sliced along the diagonal of the STO substrates (~ 45°) for FIB preparation. The distribution features of oxygen columns for both superlattice films and STO substrates are clearly displayed in the schematic along the STO-[110] axis for ABF measurements. More importantly, the quantitative analysis of the BO_6 distortions can be further performed by measuring the bond angle of *B*-O-*B*' under a simple geometrical perspective of oxygen octahedron. The basic information of the superlattice films and FIB sample for ABF-STEM measurement are shown in Supplementary Figure S12.

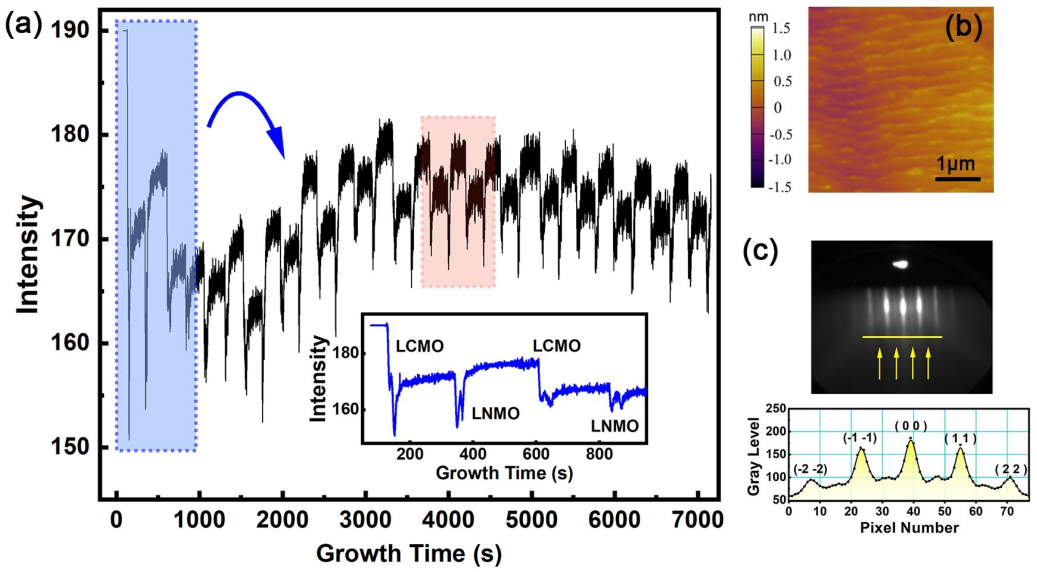

Supplementary Figure S1. Growth of La_2NiMnO_6/La_2CoMnO_6 superlattice films. **a**, Part of RHEED intensity oscillations with sixteen superlattice periods of the SL growth. The inset is a high-magnification image at initial several periods. AFM topographic images of as-grown superlattice films (**b**) shows an atomically smooth surface with step terraces. Clear additional diffraction fringes (**c**) marked by the yellow arrows are twofold superstructure peaks of double perovskites. The corresponding peaks in intensity profile are indexed as half-integer plane (k/2, k/2), where $k = \pm n$ (n = 1,3,5...).

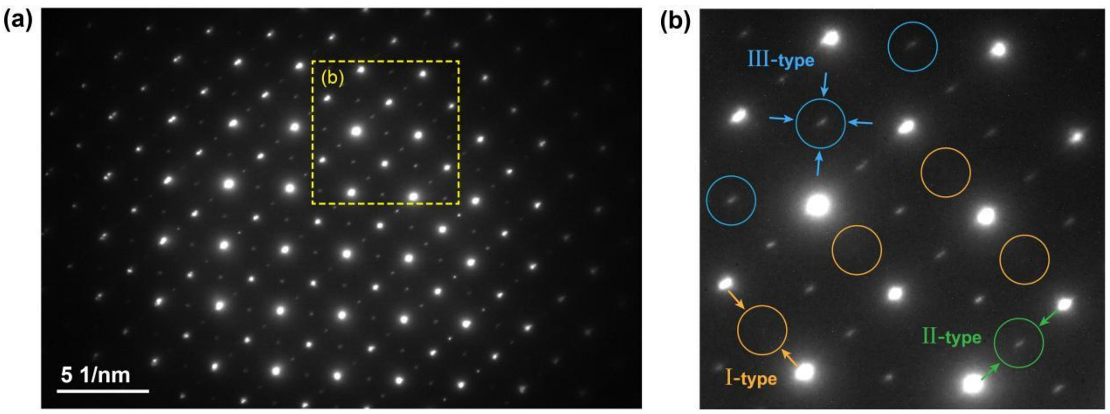

Supplementary Figure S2. Selected area electron diffraction of LNMO/LCMO superlattices with different epitaxial modes. **a**, SAED pattern of double-perovskite superlattice films SL_{90}. **b**, The local magnification image of additional diffraction spots corresponding to three types of growth modes, marked by marked by circles and arrows in different colors, respectively. The additional diffraction spots of II- and III-type are more obvious than that of I-type, which indicates a relatively few proportions of I-type epitaxial structures within the tested region.

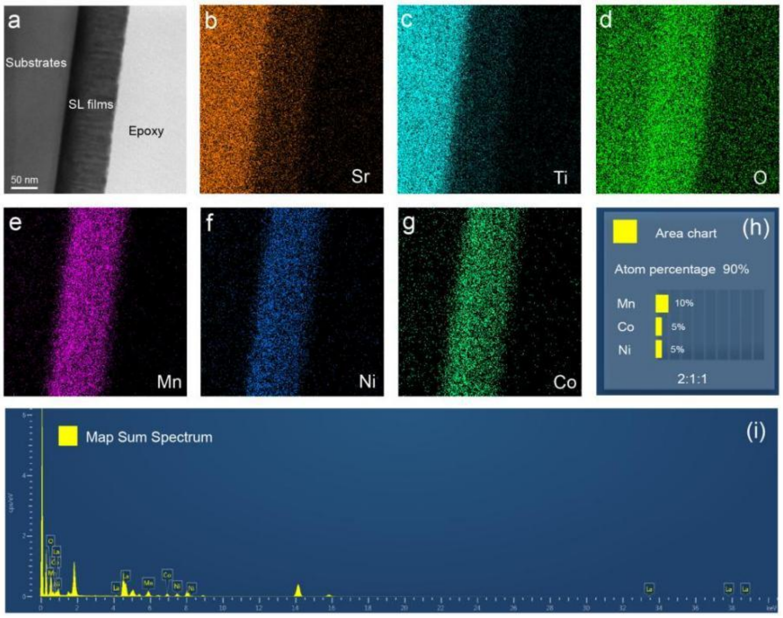

Supplementary Figure S3. The percentage of *B*-site element content for SL_{90} superlattice films. a, A low-magnification BF-STEM image of SL_{90} superlattice films on STO (001) substrate viewed along the [100] zone axis of the substrates. **b-g**, EDS-elemental mapping of Sr, Ti, O, Mn, Ni and Co for SL_{90} films, respectively. **h**, Atom percentage of Mn, Co and Ni. **i**, EDS spectrum of SL_{90}. The elemental distribution is homogeneous, with the ratio of *B*-site atoms close to 2:1:1, indicating double-perovskite superlattices with good uniformity and stoichiometry for *B*-site cations.

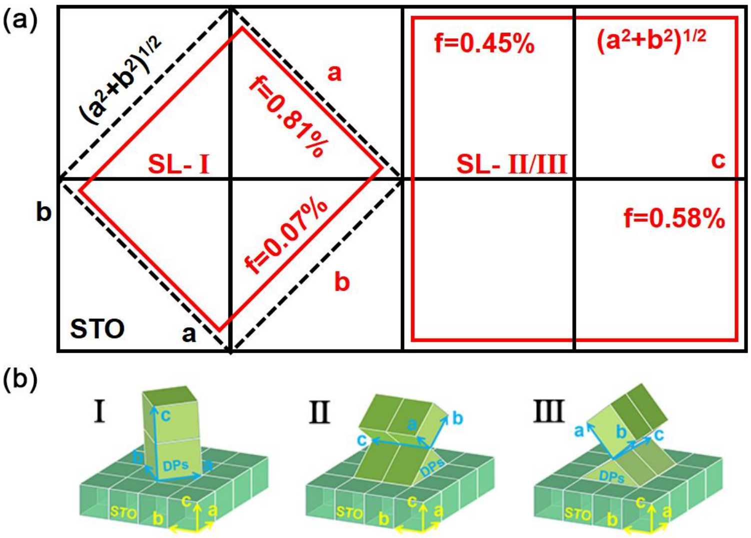

Supplementary Figure S4. Mismatch of LNMO/LCMO superlattices and STO substrates with different epitaxial modes. a, The lattice mismatch of the epitaxial modes of SL-I, SL-II and SL-III, using the formula of $f = [(d_f - d_s)/d_f]\times100\%$, where d_f and d_s are the lattice parameters of the thin films and substrates. The superlattice lattice parameters are estimated from the average of LNMO and LCMO. **b**, The three modes described by $[001]_{SL}//[001]_{Sub}$, $[001]_{SL}//[010]_{Sub}$, and $[001]_{SL}//[100]_{Sub}$.

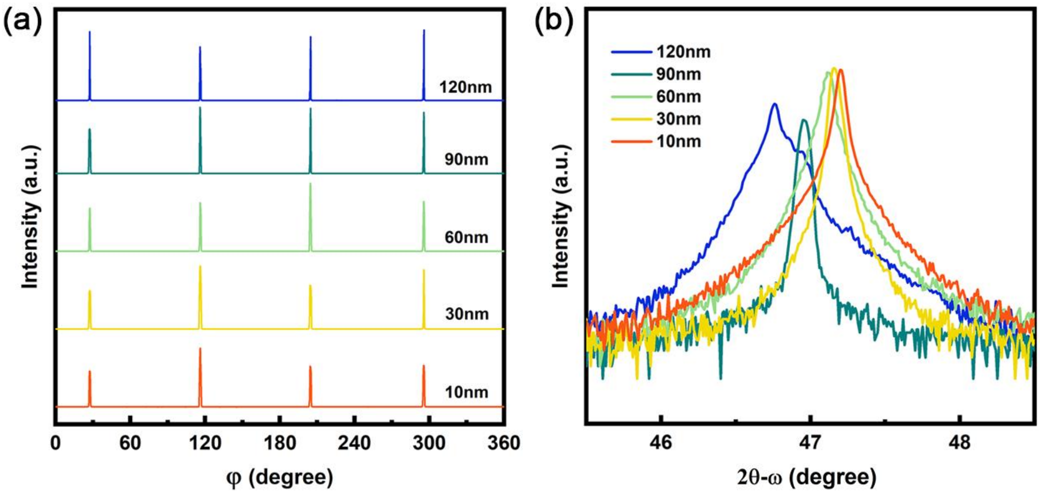

Supplementary Figure S5. The measurements of crystallinity for the superlattice films. a, The azimuthal *Φ*-scan around the (111)pc reflection of the superlattice films. The epitaxial arrangements with a fourfold rotational symmetry show the uniform crystallinity in plane. **b**, The rocking curve of (002)pc peak corresponding to the (001)-oriented substrate. The average full-width-half-maximum (FWHM) value is 0.06° indicating the high crystalline quality of the SL films. The details of FWHM with film thicknesses are displayed in Fig. 1(e) of the main text.

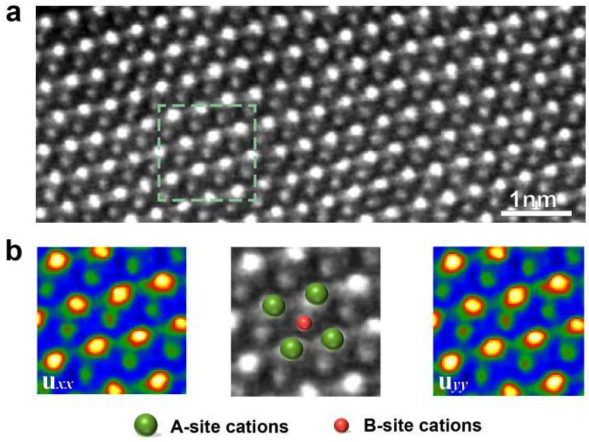

Supplementary Figure S6. Local STEM image of SL_{60}. a, STEM image of local regions at higher magnification for Figure 2d. **b**, The IP and OP atomic displacement fields (u_{xx} and u_{yy}, respectively) of local regions framed in green in (a). The green and red spheres represent *A*-site and *B*-site cations, respectively. The local atomic images with neat alignments show the epitaxial structures with coherent growth for double-perovskite superlattices.

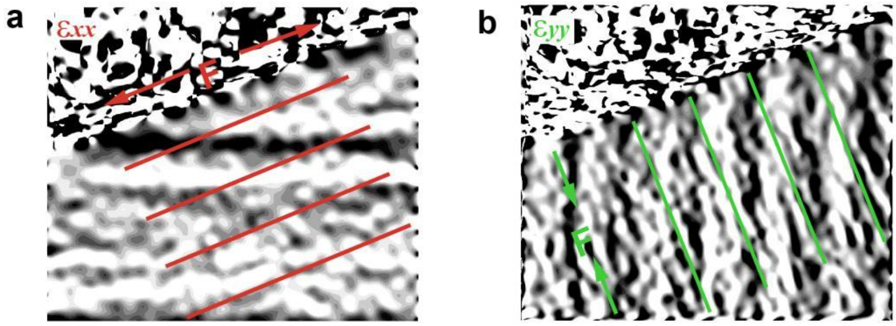

Supplementary Figure S7. Line collections for strain estimation. a, STEM image of local regions at higher magnification for Figure 2d. **b**, The IP and OP atomic displacement fields (u_{xx} and u_{yy}, respectively) of local regions framed in green in (a). The green and red spheres represent *A*-site and *B*-site cations, respectively. The local atomic images with neat.

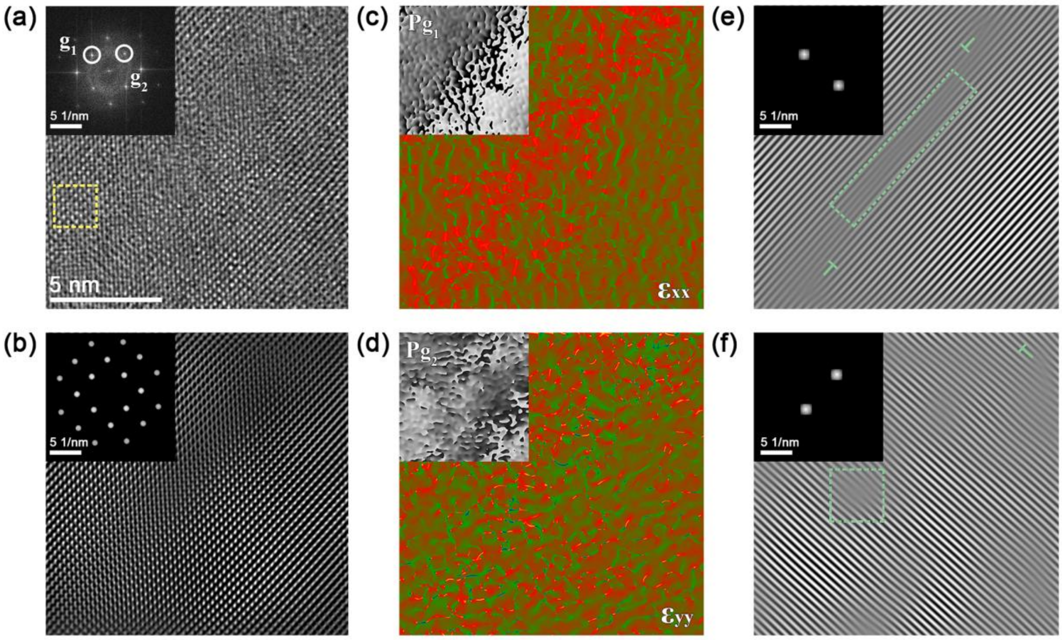

Supplementary Figure S8. Geometric phase analysis and lattice dislocations of the SL_{60} films. a, TEM image of the 60 nm thick superlattices (SL_{60}) and the corresponding FFT image. The disordered boundary of lattice registries implies the breaking of epitaxial strains within local microstructures. White circles in FFT image mark the non-collinear reciprocal lattice vectors $\mathbf{g}_1$ and $\mathbf{g}_2$ for GPA. Yellow square is the reference region for GPA. **b**, Corresponding reconstructed lattices by IFFT. **c**,**d**, GPA analysis of in-plane strain ε_{xx} (**c**) and out-of-plane strain ε_{yy} (**d**) of TEM image (**a**). The insets in $\varepsilon_{xx}/\varepsilon_{yy}$ are corresponding phase images. The strain is mainly concentrated in the disordered boundary along the direction of vector $\mathbf{g}_1$. **e**,**f**, Monochromatic filtered IFFT images of IP plane (**e**) and OP plane (**f**). Macroscopic strain is released through the formation of T-type dislocation and lattice deformation with bending fringes.

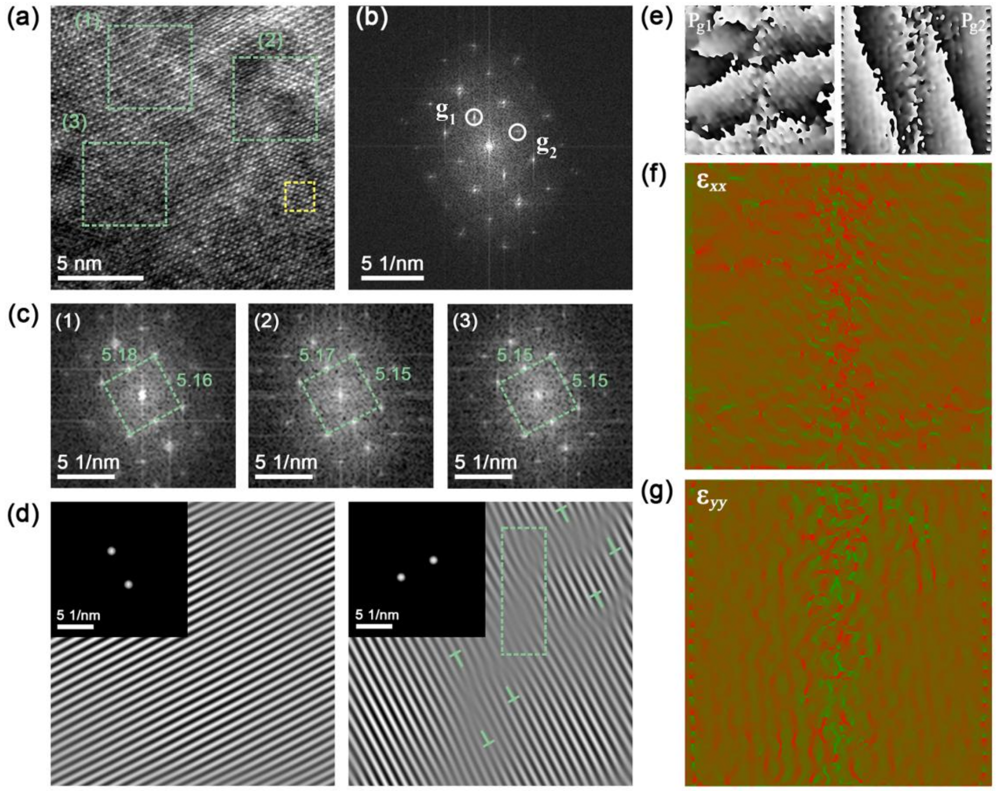

Supplementary Figure S9. Geometric phase analysis and lattice dislocations of the SL_{90} films. a,**b**, HRTEM image of the SL_{90} (**a**) and the corresponding FFT image (**b**). **c**, The FFT images of the selected areas in (**a**). The interplanar distances of OP change more obviously than that of IP, which indicates the release of local epitaxial strain with film thickness. **d**, The monochromatic filtered IFFT images of IP and OP. The IP lattice fringes are regular, while the OP lattice fringes show considerable areas with T- type dislocations and lattice deformations. **e-g**, GPA analysis of phase images (**e**) and IP strain ε_{xx} (**f**) and OP strain ε_{yy} (**g**). The non-collinear reciprocal lattice vectors $\mathbf{g}_1$ and $\mathbf{g}_2$ are selected by white circles in FFT image (**b**) for GPA. Yellow square in (**a**) is the reference region for GPA. The local strains are concentrated at the boundaries of regions (1), (2) and (3), which is consistent with the discontinuity of the output phase. These strains are originated from the dislocations in the epitaxial direction since the clamping action of the substrates decreases as the thickness.

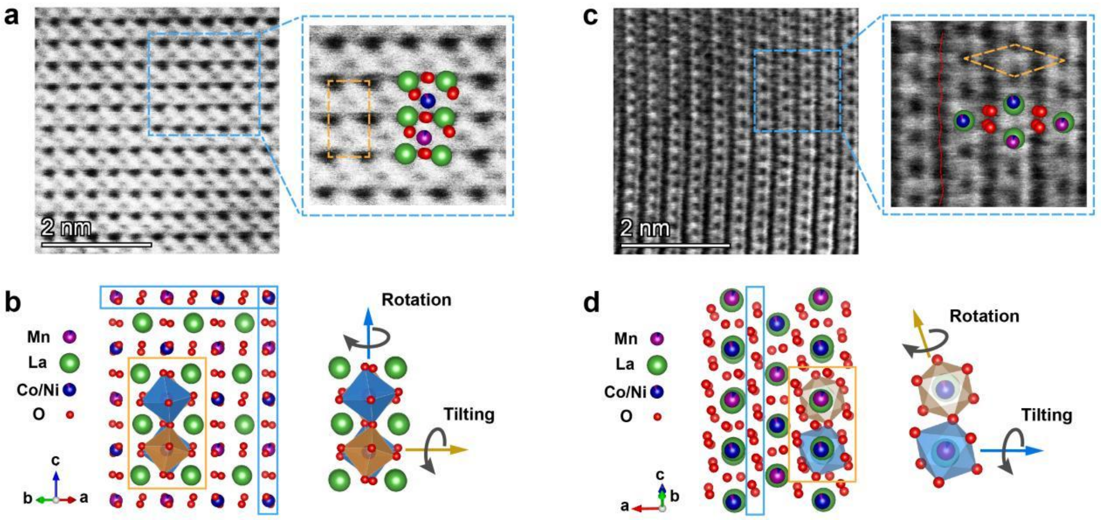

Supplementary Figure S10. Annular bright-field (ABF) STEM images of the SL_{60} films along different crystal axis directions. a,**b**, Local ABF-STEM image of SL_{60} along [110] direction (a) and the corresponding schematic of oxygen distribution (b). Due to the stacking of *A*- and *B*-site atoms with large masses, the close-packed layer structure of oxygen column is not observed in [110] direction for the SL films. Consequently, the tilting or rotation of the oxygen octahedron cannot be determined. **c,d,** ABF-STEM image with oxygen column contrast along [0 -1 1] direction for SL_{60} (c) and the corresponding oxygen distribution (d). The misaligned oxygen atoms highlighted by the curved red dashed line qualitatively indicate the oxygen octahedral distortions in the films. However, the quantitative analysis of this distortion cannot be proceeded due to the geometrical complexity of the octahedral position.

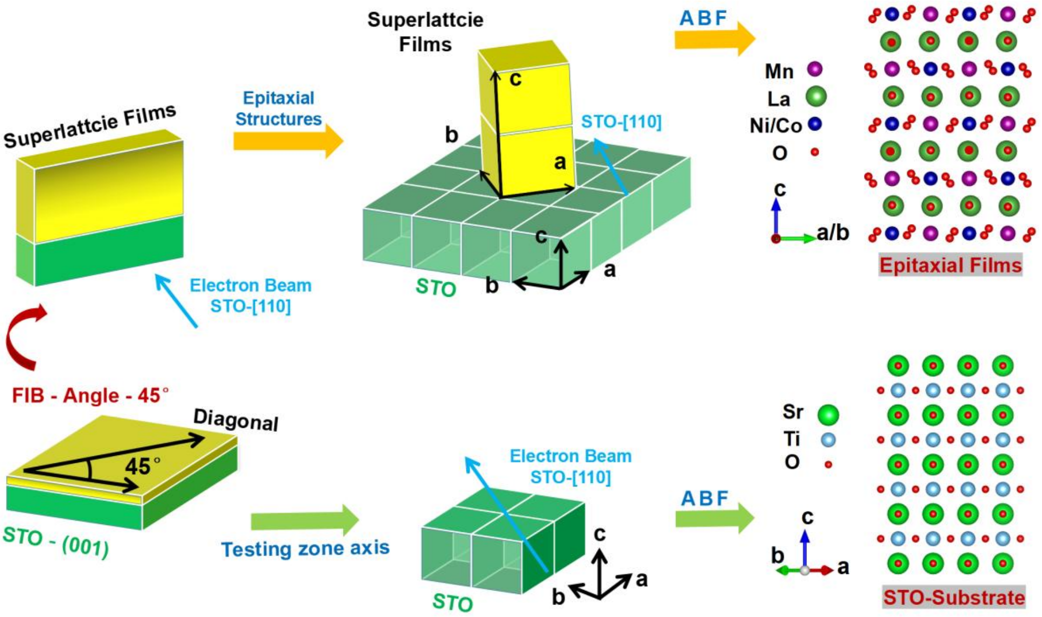

Supplementary Figure S11. The illustrations of the geometric structure for the sample preparation by using focused ion beam (FIB) and the crystal axis for the ABF measurements. The film samples are sliced parallel to the diagonal of the STO substrates (~ 45°) during the FIB preparation. Along the axis of STO-[110], the tested ABF images can clearly show the distribution feature of oxygen columns for both SL films and STO substrates. The misaligned oxygen atoms can indicate the BO_6 octahedral distortions in the films and the quantitative analysis of the distortions can be further proceeded by measuring the bond angle of *B*-O-*B*' under a simple geometrical position of oxygen octahedron.

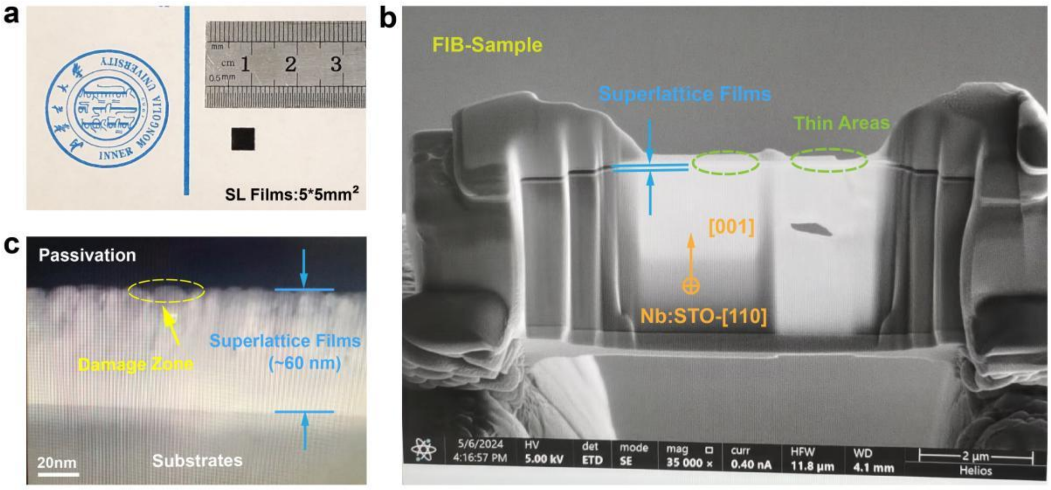

Supplementary Figure S12. The basic information of the superlattice films and FIB sample for ABF-STEM measurement. a, The superlattice films grown on (001)-Nb:STO with the size of 5×5 mm^2. **b**, Electron microscopy images of FIB samples with two thin areas at low magnification. **c**, The cross-section of SL_{60} on Nb:STO. The jagged shape of the film surface is damage zone caused by the ion beam during the FIB-sample preparation.

Supplementary Note 2: Ferroelectricity characterizations of the superlattice films with different thickness.

The profiles of PFM phase images, which are recorded along the median lines of phase images at region of 2×2 μm^2 in the Fig. 3a-c, show the corresponding phase fluctuation after writing domain (Supplementary Figure S13). The statistical values of the phase indicate the switching behavior of the spontaneous polarization under opposite electric fields. The ferroelectric macrodomains with ~180° phase contrast in the SL_{60} show the most robust ferroelectricity, which exhibits the good tunability of epitaxial strain on ferroelectricity. The most robust ferroelectricity occurs in the SL_{60} with the optimal strain rather than the largest one, implying other more intrinsic causations out of the strain mechanism for the emergence and regulation of ferroelectricity. The star-in-star pattern of domain writing with ±6 V at region of 5×5 μm^2 is then performed in the SL_{60} (Supplementary Figure S14). The clear star domains with ferroelectric phase contrast are retained for at least 3 hours after domain writing. Thus, the stable ferroelectric behavior of the SL_{60} is further confirmed.

The switching behaviors of the SL_{30} and SL_{90} are also demonstrated by writing domains (Supplementary Figure S15). While the domain distributions are poorly uniform, which stems from their weaker ferroelectricity and the differences in microstructures. In addition, the obvious phase contrast cannot be obtained in the SL_{10} and SL_{120} after writing domains (Supplementary Figure S16). On the one hand, although hybrid improper ferroelectricity is less sensitive to depolarizing fields, the ferroelectricity in relatively thin films is more difficult to characterize due to the unavoidable leakage, especially in magnetic materials with poor insulation. On the other hand, the thick films with the increased strain relaxation are unable to provide the large epitaxial strain required to induce ferroelectricity. As a result, the stable testing of ferroelectricity in superlattice films relies on the cooperative effect of both film thickness and epitaxial strain. Furthermore, we had prepared the superlattice films on the insulated $SrTiO_3$ substrates with growing $SrRuO_3$ (SRO) as the bottom electrode. The same sharp phase contrast in Supplementary Figure S17 clarifies that the nature of switched behavior originates from the ferroelectric superlattice films rather than other nonferroelectric mechanisms on Nb:STO substrates. Since the SRO buffer layer introduce new lattice mismatch for both the superlattice films and

substrates, here we adopted the SL/Nb:STO system with the epitaxial strain from single mismatch to explore the ferroelectric mechanism.

Moreover, we tested the local PFM hysteresis loops in both SL_{10} and SL_{120} films (Supplementary Figure S18). The SL_{10} films show a slight but incomplete hysteresis properties, and the SL_{120} films show no hysteresis properties. These results are consistent with the domain writing, indicating the cooperative effect of both film thickness and epitaxial strain for the stable testing of ferroelectricity in superlattice films. In order to obtain the piezoresponse (*PR*), the hysteresis loops of the first-order harmonic displacement were calculated by the formula of

$$PR(V) = A(V)\cdot \cos[\varphi(V)], \quad (1)$$

where $A(V)$ and $\varphi(V)$ are the amplitude and phase degree, respectively[3]. The diagram with applied voltage wave in SS-PFM testing and the results of the piezoresponse are shown in Supplementary Figure S19. The step triangle wave is input by function generators of direct current (dc) and alternating current (ac). The amplitude and phase signals are separated by judging the moving of laser spot. The corresponding test path is pointed out by arrows. The piezoresponse hysteresis loops represent the piezoelectric response varied with the rotation of electric dipoles[3]. For ferroelectrics, the dipole-dipole interactions can form local domain walls under continuous scanning voltage, thus the closed area of switching (S_{PR}) could also be used to estimate the ferroelectric hysteresis, expressed as

$$S_{PR} = \int [PR^{+}(V) - PR^{-}(V)]\, dV,\ V\in(-\infty,+\infty)^{4}. \quad (2)$$

The PFM hysteresis loops are subsequently measured from a 3×3 grid over a 1×1 μm^2 region of the SL_{30}, SL_{60} and SL_{90} to better explore the local ferroelectric response of the superlattice films (Supplementary Figure S20). All phase–voltage hysteresis loops, amplitude–voltage butterfly loops and piezoelectric response hysteresis loops of the nine sites in the region of 1×1 μm^2 of the superlattice films are shown in Supplementary Figures S21-S23. The distribution of piezoresponse in general indicates a large tuning of the ferroelectricity by the epitaxial strain. Furthermore, the bias imprints of all films are negative, indicating the downward spontaneous polarization guided by the surface polarity of Nb-doped substrates (Supplementary Figure S24). The imprint of the SL_{60} is the smallest because the strong depolarization

field (E_{dep}) from ferroelectricity can help to reduce the influence of the built-in electric field (E_{in}) on the testing.

The corresponding integrated current of the SL_{60} films shows typical double peaks of polarization switching as a function of the applied voltage (Supplementary Figure S25). This result is credible evidence for the polarization switching in ferroelectrics and demonstrates the intrinsic nature of the ferroelectricity in the superlattice films. The polarization and coercive field of *P*-*E* hysteresis loops for all films with different thicknesses further indicate the effective regulation of the ferroelectricity by the epitaxial strain. Although the *P*-*E* hysteresis loops measured by PUND method has shown the remanent polarization, it can hardly be measured by the traditional method because of the differences of local leakage and polarization in the films. Thus, we built a ferroelectric probe test bench with reference to the contact mode of the PFM conductive probe[5]. According to the qualitative test by using the self-built probe station, the ferroelectricity of the SL_{60} is the strongest, and the polarization ratio of the films is similar to the result of PUND measurements (Supplementary Figure S26). The coercive fields of the *P*-*E* hysteresis loops detected by PFM tip are much larger than that in PUND due to the differences in two physical processes during the tests.

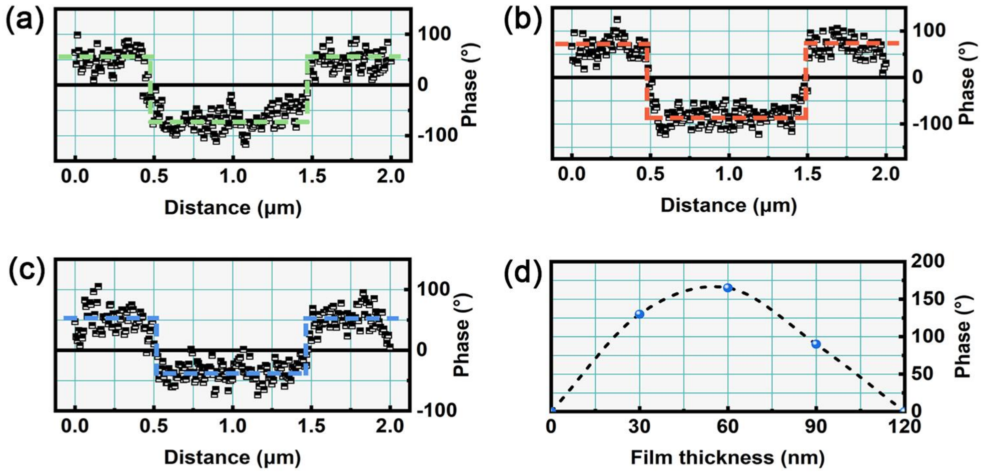

Supplementary Figure S13. The line profiles of the switching phase. a-c, The profiles of the corresponding phase fluctuation of PFM phase images recorded along the median lines of phase images after writing domain at region of 2×2 μm^2 in the Figs. 3(a)-3(c). **d**, Statistical values of the phase.

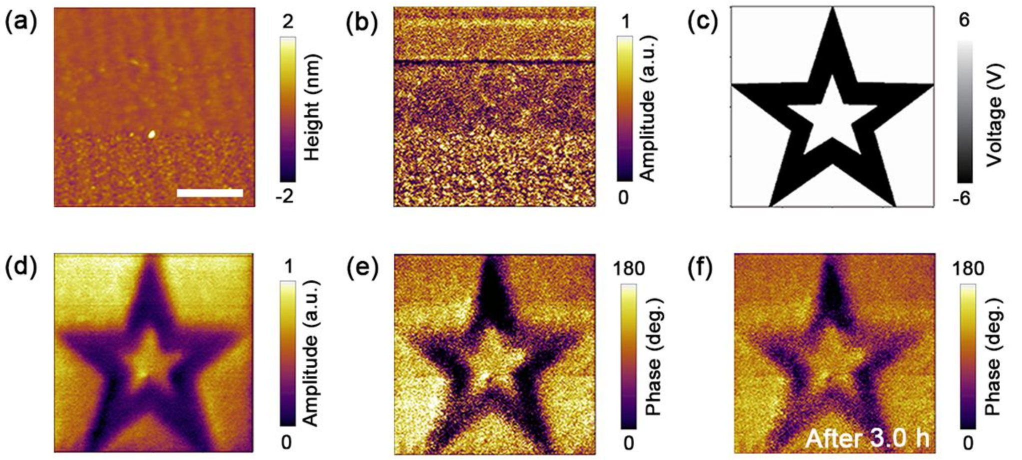

Supplementary Figure S14. The room-temperature ferroelectricity of the SL_{60} superlattice films characterized by ferroelectric domain writing/retention measurements. a,**b**, The topography image (**a**) and initial PFM amplitude image (**b**) of the SL_{60} at region of 5×5 μm^2. The scale bar in topography image is 2.0 μm. **c**, The star-in-star pattern with ±6 V for domain writing. **d**,**e**, The PFM amplitude image (**d**) and phase image (**e**) after domain writing. **f**, The PFM phase image after writing 3.0 h. The domain patterns are written by a biased conductive tip.

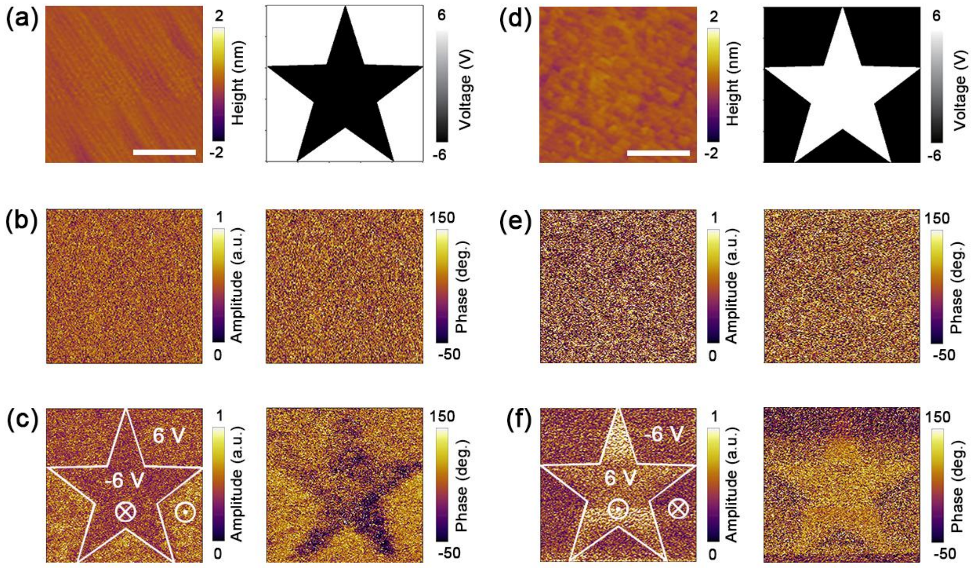

Supplementary Figure S15. Writing domain patterns of the SL_{30} and SL_{90} films in the region of 5×5 μm^2. a, The topography image and writing mode with ±6V star-in-box pattern of the SL_{30} films. **b**,**c**, Corresponding PFM amplitude and phase images before (**b**) and after (**c**) writing domain. **d**, The topography image and star-in-box pattern of the SL_{120} films. **e**,**f**, Corresponding PFM amplitude and phase images before (**e**) and after (**f**) writing domain. All scale bars are 2.0 μm. The writing domain tests at large regions indicate macroscopic ferroelectricity in both SL_{30} and SL_{90} films, while the ferroelectricity in the SL_{30} films is more robust than that in the SL_{90} films.

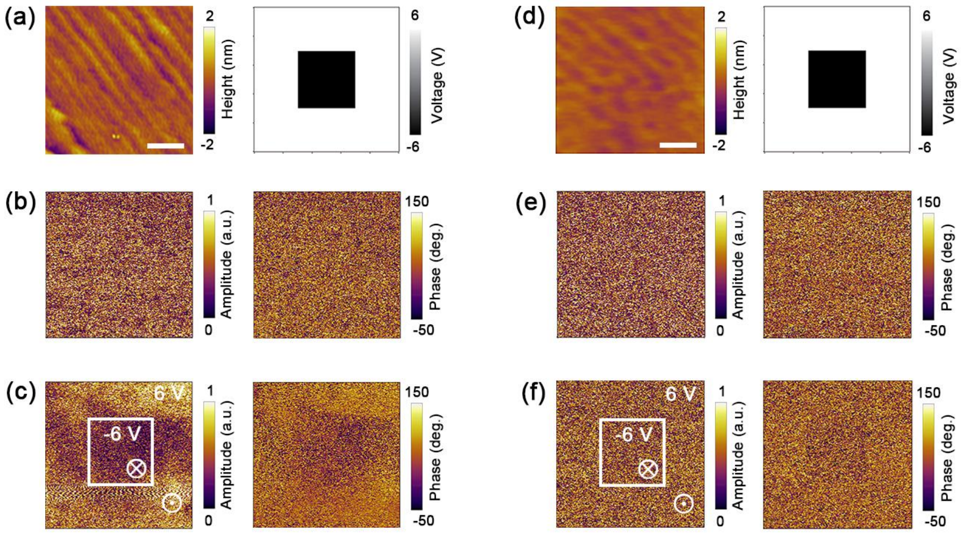

Supplementary Figure S16. Writing domain patterns of the SL_{10} and SL_{120} films. **a**, The topography image and writing mode with ±6V box-in-box pattern of the SL_{10} films. **b**,**c**, Corresponding PFM amplitude and phase images before (**b**) and after (**c**) writing domain. **d**, The topography image and box-in-box pattern of the SL_{120} films. **e**,**f**, Corresponding PFM amplitude and phase images before (**e**) and after (**f**) writing domain. All scale bars are 0.5 μm. The SL_{10} films without a phase contrast of two boxes show less stable ferroelectricity. On the one hand, a few superlattice periods cannot allow improper ferroelectricity to form large-sized domains. On the other hand, excessive strains seem to inhibit such ferroelectricity. The SL_{120} films show no ferroelectricity due to strain release.

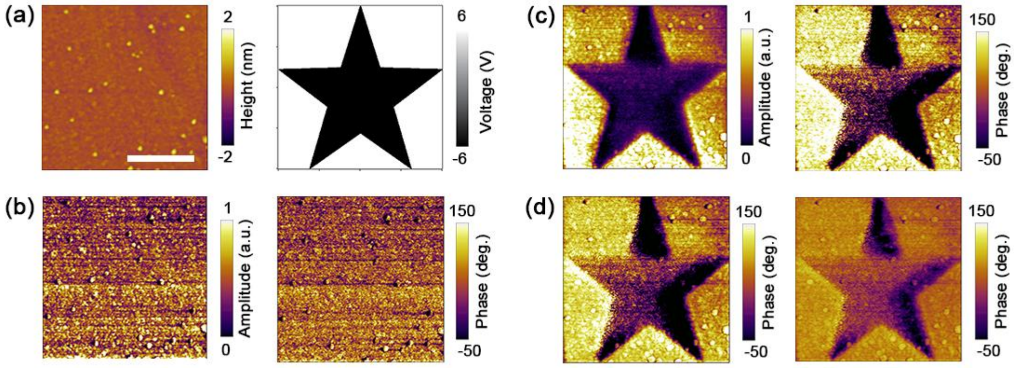

Supplementary Figure S17. Writing domain patterns of the superlattice films with $SrRuO_3$ as the bottom electrode. **a**, The topography image and writing mode with ±6V star-in-box patterns. **b**,**c**, Corresponding PFM amplitude and phase images before (**b**) and after (**c**) writing domain. (**d**) The phase images of writing domain after 0.5 and 1.0 h. The scale bar is 2.0 μm. Classical phase contrast of retention characteristics (**d**) indicates that the robust ferroelectricity originates from the ferroelectric superlattice films rather than other nonferroelectric mechanisms on Nb:STO substrates. Since the SRO buffer layer introduce new lattice mismatch for both the superlattice films and substrates, here we adopted the SL/Nb:STO system with the epitaxial strain from single mismatch to explore the ferroelectric mechanism.

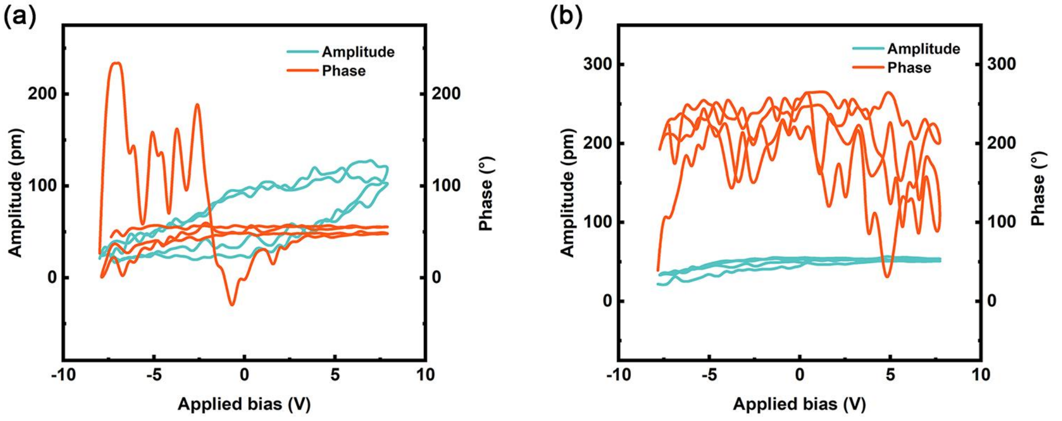

Supplementary Figure S18. The SS-PFM testing for SL_{10} and SL_{120} films. a,**b**, SS-PFM testing of the SL_{10} (**a**) and SL_{120} (**b**) films. The SL_{10} films show a slight but incomplete hysteresis properties, indicating unstable ferroelectricity as described in writing domain test. In the result of (**b**), the SL_{120} films show no hysteresis properties because of the strain release with increasing film thicknesses.

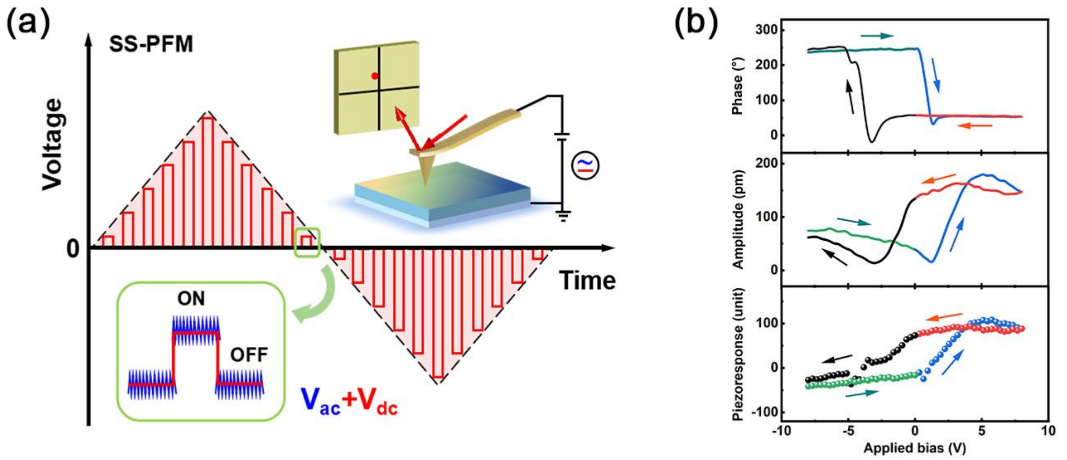

Supplementary Figure S19. The testing schematic of switching spectroscopy PFM (SS-PFM). a, The diagram with applied voltage wave in SS-PFM testing. The step triangle wave is input by function generators of direct current (dc) and alternating current (ac). The amplitude and phase signals are separated by judging the moving of laser spot. **b**, phase switching hysteresis loop (φ), amplitude butterfly loop (*A*) and piezoelectric response (*PR*) hysteresis loop (*A*cosφ) of a typical ferroelectric material in SS-PFM testing (from top to bottom). The corresponding test path is pointed out by arrows.

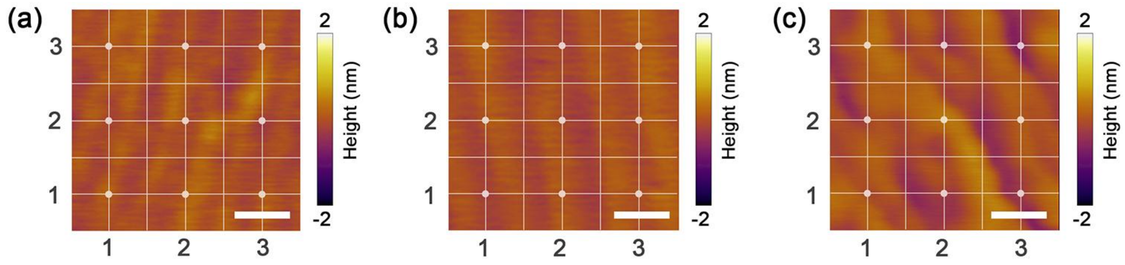

Supplementary Figure S20. The topography images for SS-PFM testing. a-c, Local topography regions for testing piezoelectric response distributions of the SL_{30} (**a**), SL_{60} (**b**) and SL_{90} (**c**), respectively. The scale bar is 0.25 μm.

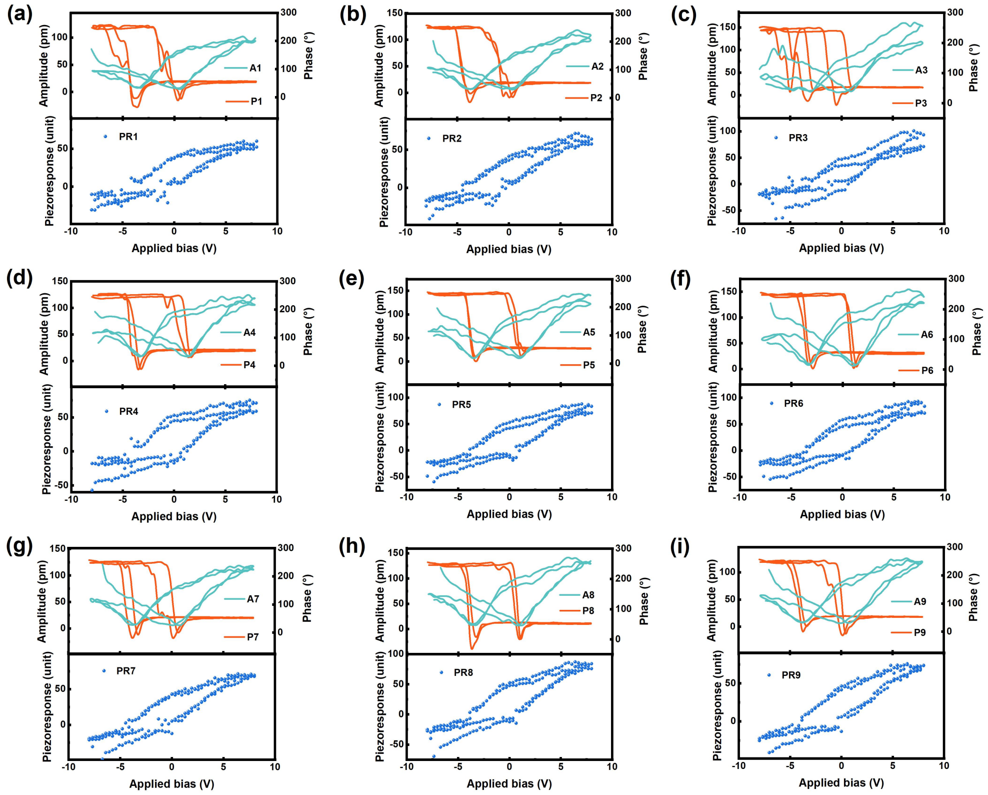

Supplementary Figure S21. SS-PFM testing for SL_{30} films. a-i All phase–voltage hysteresis loops, amplitude–voltage butterfly loops and piezoelectric response hysteresis loops of the nine sites in the region of 1×1 μm^2 of the SL_{30} films as displayed in Supplementary Figure S20a, respectively.

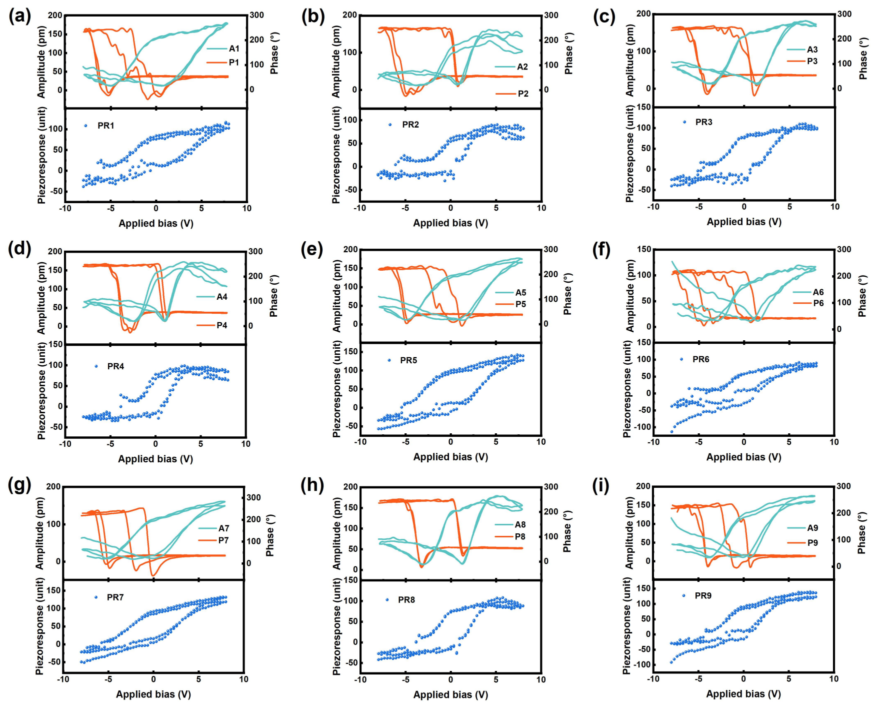

Supplementary Figure S22. SS-PFM testing for SL_{60} films. a-i All phase–voltage hysteresis loops, amplitude–voltage butterfly loops and piezoelectric response hysteresis loops of the nine sites in the region of 1×1 μm^2 of the SL_{60} films as displayed in Supplementary Figure S20b, respectively.

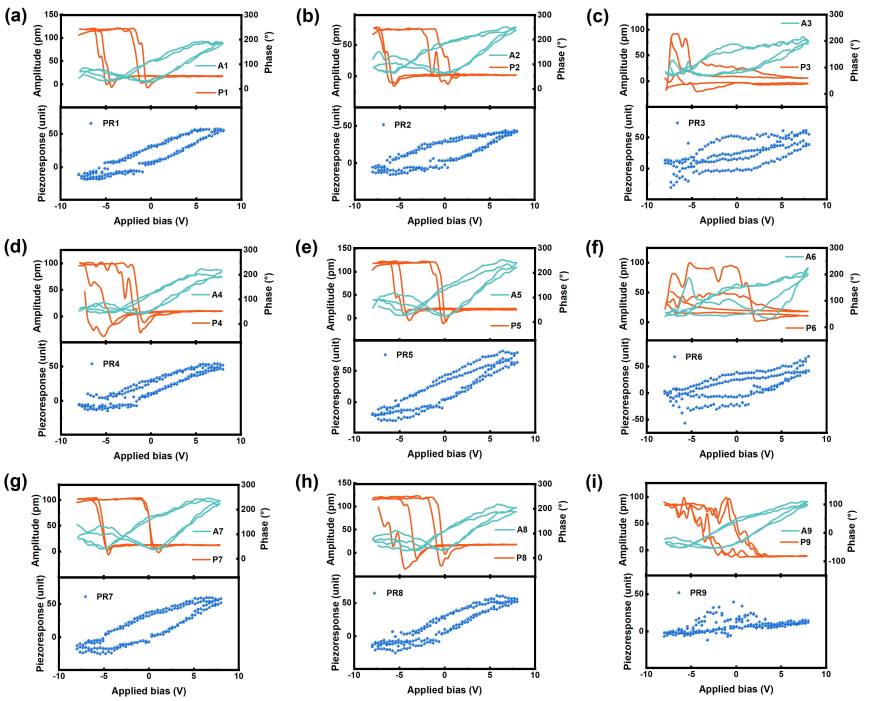

Supplementary Figure S23. SS-PFM testing for SL_{90} films. a-i All phase–voltage hysteresis loops, amplitude–voltage butterfly loops and piezoelectric response hysteresis loops of the nine sites in the region of 1×1 μm^2 of the SL_{90} films as displayed in Supplementary Figure S20c, respectively.

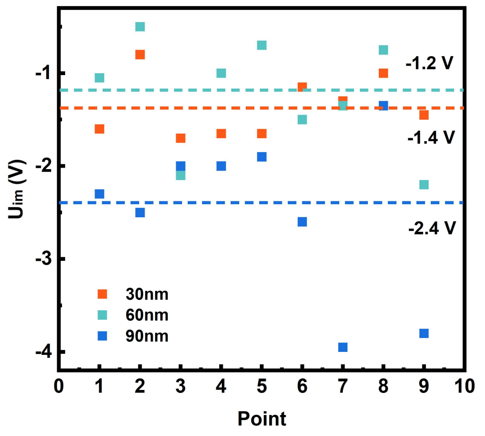

Supplementary Figure S24. Bias imprint (U_{im}) of coercive voltages for the SL_{30}, SL_{60} and SL_{90} films. The imprint of the SL_{60} is the smallest, which indicates the strong depolarization field (E_{dep}) from ferroelectricity can help to reduce the influence of the built-in electric field (E_{in}) on the testing.

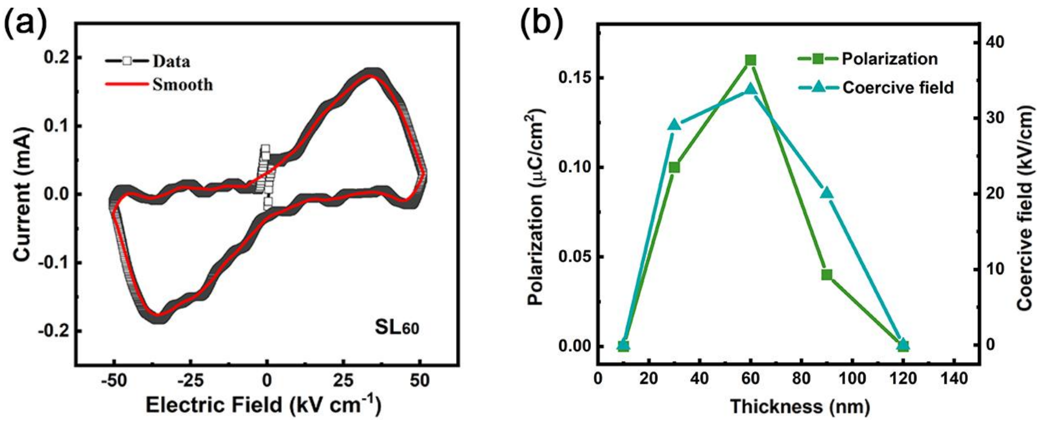

Supplementary Figure S25. Ferroelectric characterization and numerical statistics. a, The corresponding integrated current of the SL_{60} films as a function of the applied voltage. **b**, The polarization and coercive field of *P*-*E* hysteresis loops (PUND testing) for the superlattice films with different thicknesses. The ferroelectricity of the SL_{60} films is the strongest.

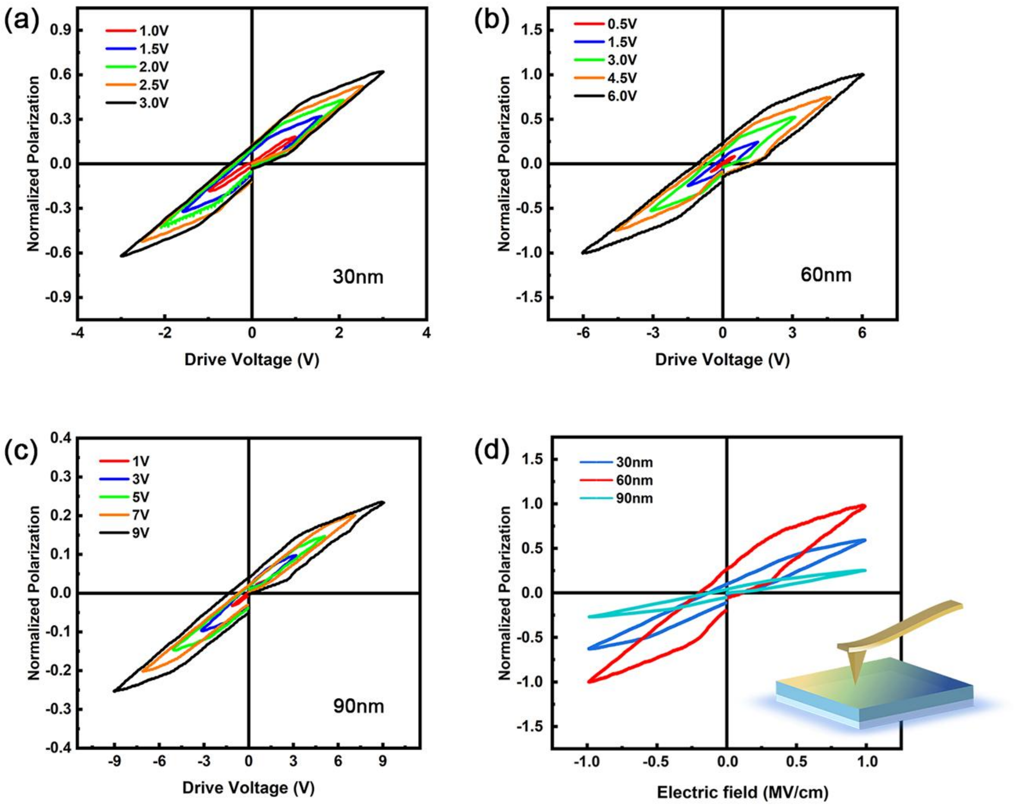

Supplementary Figure S26. The qualitative measurements of ferroelectric hysteresis loop using the self-built probe station. a-c, *P-E* hysteresis loops of the SL_{30} (**a**), SL_{60} (**b**) and SL_{90} (**c**), respectively. **d**, The statistics of *P-E* hysteresis loops for the films with different thicknesses measured by self-built probe station. The inset is the positional diagrams of the films and probe during test. The relative polarization is normalized by the SL_{60} with maximum polarization value. The details of the self-built probe station had been introduced in previous study.

Supplementary Note 3: Estimation of piezoelectric coefficient (d_{33}).

In this section, we combine the dielectric theory approximation and PFM experimental testing to estimate the piezoelectric coefficient (d_{33}) of the samples. As shown in Supplementary Figure S19, the SPM tip is applied with a triangle−square waveform when the testing of switching spectroscopy PFM is operated. The time for bias-on and bias-off is 25 ms, respectively, to obtain the bias-on and bias-off phase hysteresis loops (φ) and amplitude loops (A). The piezoelectric hysteresis loop (PR) can be calculated from the equation[4]:

$$PR = A\cos\varphi. \tag{3}$$

The average piezoelectric response from the loop is obtained by

$$PR = (|PR^{+}| + |PR^{-}|)\ /\ 2. \tag{4}$$

According to the definition of piezoelectric coefficient, the estimated value of d_{33} can be calculated from the derivative of the PFM butterfly curve by the formula:

$$d'_{33} = f_0\,|_{\mathrm{d}f/\mathrm{d}U = 0},$$

$$f = PR/U_{\mathrm{bias}}. \tag{5}$$

The curve (f vs U_{bias}) is displayed in Supplementary Figure S27. However, the piezoelectric response from PFM is not the direct displacement of the sample for PR. It is necessary for PFM test to be calibrated by the displacement on a standard sample, such as quartz or $LiNbO_3$. The relatively accurate PR' is then referenced by[6]

$$PR' = PR/G, \tag{6}$$

where G is the gain of the equipment (PFM) during the measurement. Since the testing parameters are the same for all films, the proportional relationship of d_{33} can be estimated without the certain calibration as

$$d'_{33(\mathrm{SL}\text{-}90)} : d'_{33(\mathrm{SL}\text{-}60)} : d'_{33(\mathrm{SL}\text{-}30)} = 1 : 2.26 : 1.63.$$

On the other hand, the well-known phenomenological relation for d_{33} is[7, 8]

$$d_{33\text{-phe}} = 2Q_{elec}\,\varepsilon_0\varepsilon_r P, \tag{7}$$

where Q_{elec} is the electrostrictive coefficient, ε_0 is the permittivity of vacuum, ε_r is the relative permittivity, and P is the spontaneous polarization of the sample. According to the piezoelectric effect, the corresponding strain ε_{33} can be calculated by[9]

$$\varepsilon_{33} = d_{33\text{-phe}}E. \tag{8}$$

When an external electric field is applied, the strain of the films should also include the electrostrictive term (ε_Q), which can be written as[10]

$$\varepsilon_Q = Q_{elec} P^2. \tag{9}$$

Thus, for a system where the spontaneous polarization is tested by applying an external electric field, the overall strain ε in thin films can be expressed as

$$\varepsilon = \varepsilon_{33} + \varepsilon_Q + k = Q_{elec} P^2 + Q_m P + k_0, \tag{10}$$

where $Q_m = 2\varepsilon_0\varepsilon_r Q_{elec} E$, and k_0 is the simplified higher-order small quantity related to the other effect of polarization reversal.

In this system, the spontaneous polarization is downward guided by the surface polarity of the substrates, and different strains correspond to different ferroelectric polarization one by one. Therefore, the phenomenological result of all films can be approximated as the testing process with different electric field for one ferroelectric sample, since the only excitation of ferroelectricity in the system arises from strain. As shown in Supplementary Figure S28a, the approximate Q_{elec} is fitted as $(4.97 \pm 0.91)\times10^{-3}$ m^4/C^2. Eventually, the piezoelectric coefficient (d_{33}) is estimated by

$$d_{33} = d_{33\text{-hpe}} \cdot d'_{33\text{-SL}}. \tag{11}$$

The relative permittivity ε_r is shown in Supplementary Figure S28b. The estimated d_{33} for the SL_{30}, SL_{60} and SL_{90} films are 0.9, 2.0, and 0.2 pm/V, respectively. The coefficient Q_m calculated from the d_{33} is on the same order of magnitude with the fitting value, which indicates the approximate method for estimating d_{33} is reasonable. The estimated proportion and result were plotted in Figure 3k.

To explore the regulation of strain on ferroelectricity more clearly, we defined the tuning gain (η), described as

$$\eta_{x\text{-}y} = (R_y / R_x)\times 100\%, \tag{12}$$

where R_x and R_y are the variation range of effective excitation (x) and response (y) signals for a physical process, respectively, and the variation range is denoted as

$$R_x = [(x_{\max} - x_{\min})/|x_{\min}|]\times 100\%. \tag{13}$$

When $\eta > 1$, the tuning of the excitation to the response corresponds to a process of signal amplification; while for $\eta < 1$, it corresponds to an attenuation process. In this system, the tuning gain of strain to polarization $\eta_{\varepsilon\text{-}p}$ reaches ~280% (where $R_\varepsilon \approx 105\%$, $R_p \approx 300\%$), which concretely indicates the large tuning of epitaxial strain on ferroelectricity.

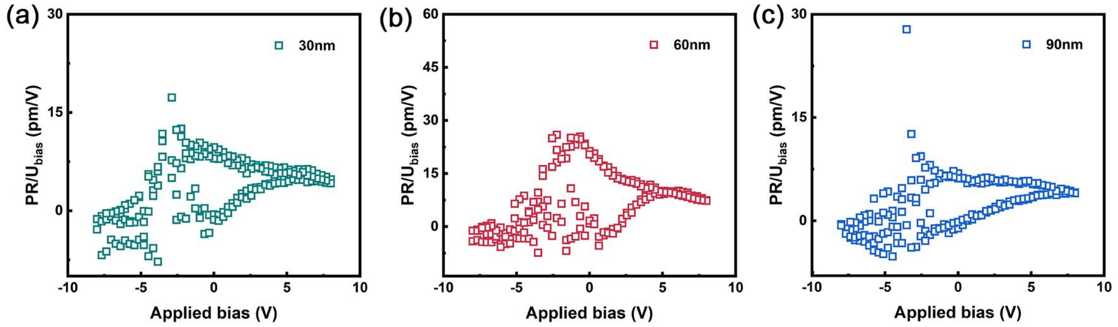

Supplementary Figure S27. The estimated piezoelectric coefficient of different thickness films. a-c, Piezoelectric response hysteresis loops (PR/U_{bias}-U_{bias}) of the SL_{30}, SL_{60} and SL_{90} films, respectively.

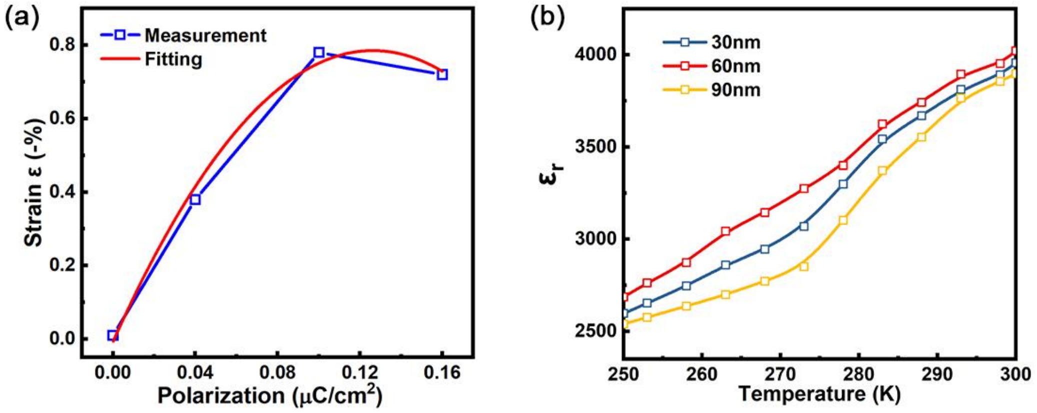

Supplementary Figure S28. The estimated electrostrictive coefficient and dielectric properties. a, The *P-ε* curve to estimated electrostrictive coefficient of SL films by fitting function. The estimated value is $(7.72 \pm 0.74)\times10^{-3}$ m^4/C^2. **b**, The relative dielectric constant of the SL_{30}, SL_{60} and SL_{90} films for estimating piezoelectric coefficient.

Supplementary Note 4: X-ray photoelectron spectroscopy and Raman spectra characterizations.

Since the 2*p* core-level X-ray photoelectron spectroscopy (XPS) of transition metal ions is sensitive to the 3*d*-electron content[11], XPS has become a useful tool to analyze the surface information of chemical composition and elemental oxidation state. Supplementary Figure S29 shows the XPS core-level spectra of Mn 2*p* for La_2NiMnO_6/La_2CoMnO_6 superlattice films with different thicknesses. All core-level peaks are charge calibrated by C1*s* peak (284.8 eV), and the peak fitting adopts Gaussian-Lorentzian method and Shirley background analysis. The Mn 2*p*3/2 and Mn 2*p*1/2 peaks at the binding energies (BEs) are about 641 and 653 eV, which can be distinguished from the BEs of 641.1 and 642.6 eV in Mn_2O_3 and MnO_2, respectively[12]. The corresponding fitted peaks and percentages of Mn^{4+} and Mn^{3+} ions were listed in Supplementary Table S1. The percentages of Mn^{4+} are almost 90%, which indicates the valence state of Mn ions is unchanged with film thickness. This result further ruled out that the difference in ferroelectricity between the superlattice films does not stem from the Jahn-Teller effect of Mn^{3+} ions. Notably, all peaks of Mn $2p_{3/2}$ and Mn $2p_{1/2}$ at BEs differ by 11.5 (±0.1) eV and the $2p_{3/2}$ peak is about twice the area of $2p_{1/2}$ peak, which guarantees the accuracy of fitted peaks. More importantly, the valence state of Ni cannot be directly obtained due to the virtue of the strong overlap of Ni $2p_{3/2}$ peak and La $3d_{3/2}$ peak for XPS[13]. The ion pairs of Mn^{4+}/Co^{2+} and Mn^{4+}/Ni^{2+} (Mn^{3+}/Co^{3+} and Mn^{3+}/Ni^{3+}) occur in the double-perovskite films, corresponding to the high (low) oxidation state. Therefore, the level of the mixed valence states of all transition-metal ions in superlattice films can be assessed by the oxidation state of Mn ions. Furthermore, the excellent oxygen stoichiometry for double-perovskite superlattice films is benefited from the ozone depositing and annealing processes.

Raman spectroscopy is an effective approach to analyze the information of local microstructures and lattice distortions, through probing Raman-active modes at the Brillouin zone center from the changes of crystal symmetry[14, 15]. Raman spectrum with wide wavenumber range of the SL_{30}, SL_{60} and SL_{90} films originate from the crystal structure of double perovskites (Supplementary Figure S30). The peaks of 530 and 650 cm^{-1} correspond the antisymmetric stretching (AS) and symmetric stretching

(S) vibration modes of BO_6 octahedron in double-perovskite La_2NiMnO_6/La_2CoMnO_6 superlattices[16]. Another two weak scattering peaks around high frequency (1000-1400 cm^{-1}) appear to be associated with two-phonon scattering[17]. Such vibration modes are related to combination and overtone modes of fundamental stretching modes. Compared to the substrate peaks without shift, the peaks of Raman vibration modes shift respectively with film thickness, which is related to the different oxygen octahedral distortion modes driven by epitaxial strain. More interestingly, these changes in Raman vibration modes reflect the different sensitive between antisymmetric and symmetric stretching modes to the biaxial strains or compressive/tensile strains in the lattice structures. It can been also found that the S vibration modes of all superlattice films exhibit a similar narrow linewidth with FWHM ≈ 53 cm^{-1}, indicating the high B-site ordering in double-perovskite superlattices.

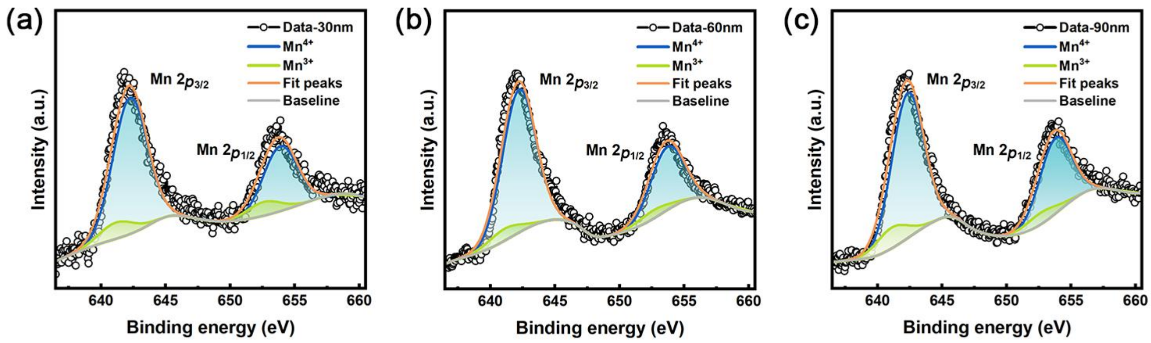

Supplementary Figure S29. X-ray photoelectron spectroscopy (XPS) analysis. a-c, XPS core-level spectra of Mn 2p for La_2NiMnO_6/La_2CoMnO_6 superlattice films with different thicknesses of 30, 60, and 90 nm, respectively.

Supplementary Table S1. The fitted peaks and percentages of the Mn 2*p* from XPS Data.

element	$2p_{3/2}$ (eV)	$2p_{1/2}$ (eV)	percentage (%)
Mn-SL_{30}	642.1	653.7	-
Mn^{3+}	641.2	652.6	11
Mn^{4+}	642.2	653.9	89
Mn-SL_{60}	642.1	653.6	-
Mn^{3+}	641.1	652.6	10
Mn^{4+}	642.2	653.7	90
Mn-SL_{90}	642.2	653.7	-
Mn^{3+}	641.1	652.7	12
Mn^{4+}	642.3	653.8	88

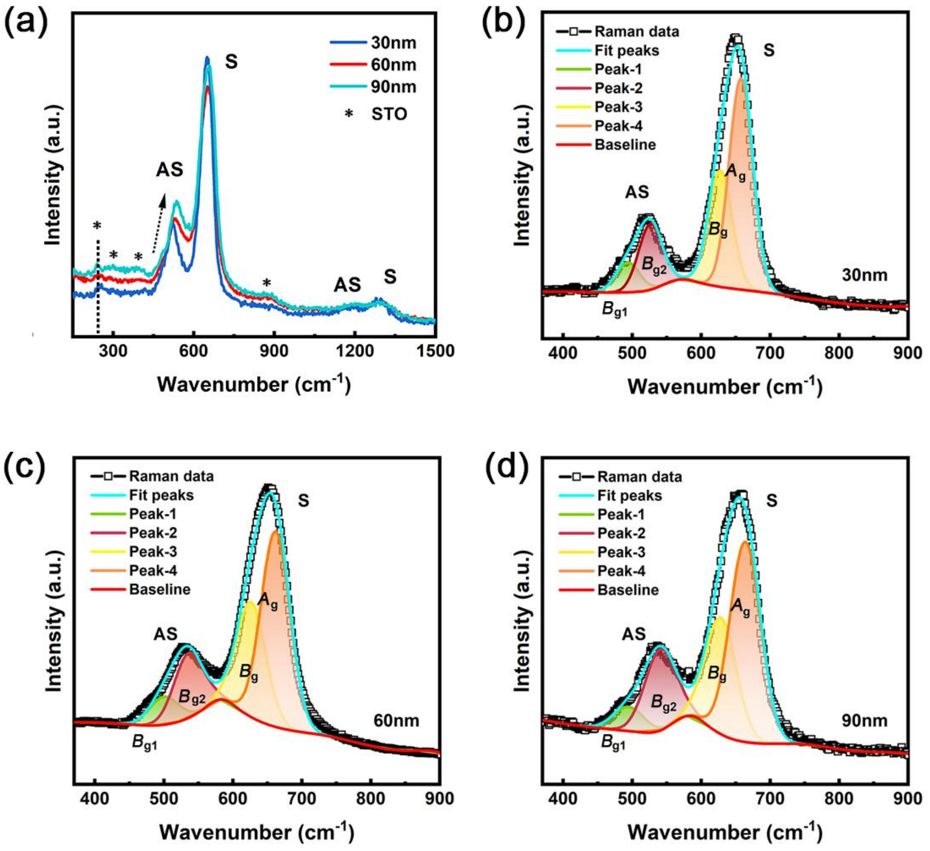

Supplementary Figure S30. Raman spectra testing of the superlattice films. a, Raman spectrum with wide wavenumber range of the SL_{30}, SL_{60} and SL_{90} films. **b-d**, The Raman fitting peaks of the SL_{30} (**b**), SL_{60} (**c**) and SL_{90} (**d**).

Supplementary Note 5: DFT simulations for octahedral distortion.

In this Supplementary Note, we introduce a labeling method of atomic site (A_{ijk}), *B*-O bond length (L_{ijk}), and *B*-O-*B*' bond angle (Φ_{ijk}) for double-perovskite crystal structure, as shown in Supplementary Figure S31. In this method, the determination of bond length and bond angle is based on the mark of atomic sites. A_{ij} represents *B*-site cation, where *i* represents the located plane for the marked cation, and *j* represents the specific cation site in the plane. Thus, A_{ijk} represents the oxygen ion around the cation A_{ij}, where *k* represents the specific oxygen ion site. Thereby, L_{ijk} represents the bond lengths of *B*-O between the *B*-site cation A_{ij} and the oxygen ion A_{ijk}. By analogy, Φ_{ijk} represents the *B*-O-*B*' bond angle based on the cation A_{ij}, where *k* represents the orientation of another *B*-site cation to form a bond angle with the cation A_{ij}. All the information about the location and direction is clearly stated in the schematic. In short, the individual ions, bond lengths and bond angles in the double-perovskite crystal structures can be conveniently described as A_{ij}/A_{ijk}, L_{ijk}, and Φ_{ijk}, using this labeling method.

The differences in BO_6 oxygen octahedral distortion driven by strain are further confirmed through the DFT calculation in the Castep model of Materials Studio. The corresponding details of optimized superlattice structures under the equivalent strain are listed in Supplementary Tables S2 and S3 using the above labeling method. According to the structural statistics, the magnitude of the oxygen octahedral distortions in superlattice films with different epitaxial strains are significantly different, while the distortion modes are almost the same, as shown by the schematic in Figure 4d. The trends in the magnitude of the octahedral distortions concerning the bond length and bond angle for the films with different epitaxial strain are clearly shown as a function of film thicknesses (Supplementary Figure S32). For the double-perovskite crystals with out-of-plane compressive strain and in-plane tensile strain, the corresponding *B*-O-*B*' bond angle and *B*-O bond length are closely associated with oxygen octahedral rotation (OOR) and titling (OOT). As usual for hybrid improper ferroelectrics under compressive strain, OOR is enhanced, while OOT is suppressed[18]. Therefore, the most robust ferroelectricity occurs in SL_{60} with the strongest coupling of OOR and OOT around the critical strain, corresponding to

the sudden changes in the octahedral distortion. Meanwhile, the optimal coupling state of OOR and OOT generates a higher stability of the ferroelectric structure.

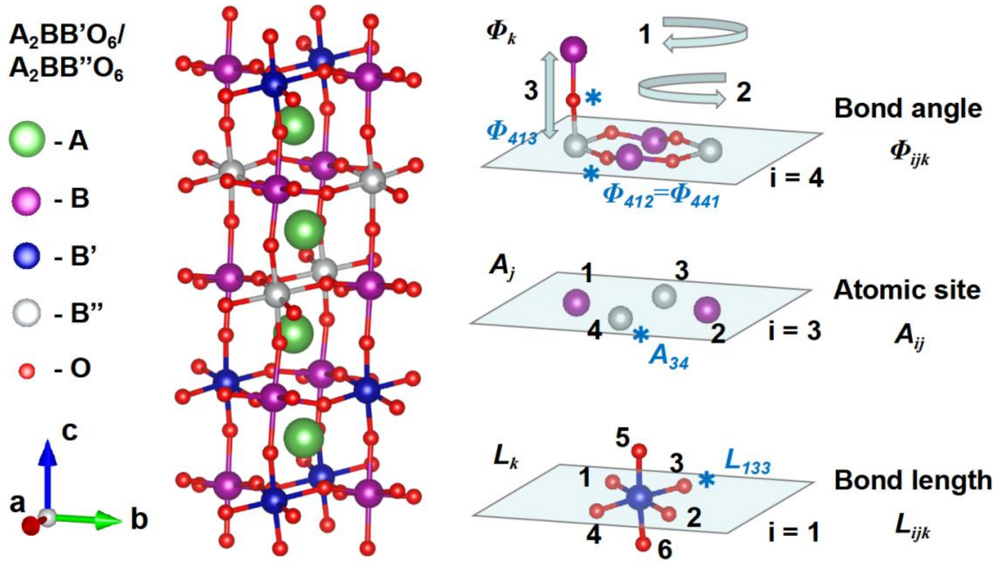

Supplementary Figure S31. The labeling method of atomic site (A_{ijk}), *B*-O bond length (L_{ijk}) and *B*-O-*B* bond angle (Φ_{ijk}) of double-perovskite superlattice unit cell. In this method, the determination of bond length and bond angle is based on the mark of atomic sites. All labeled bond lengths and bond angles correspond to the nearest neighbor composition of the determined atomic site.

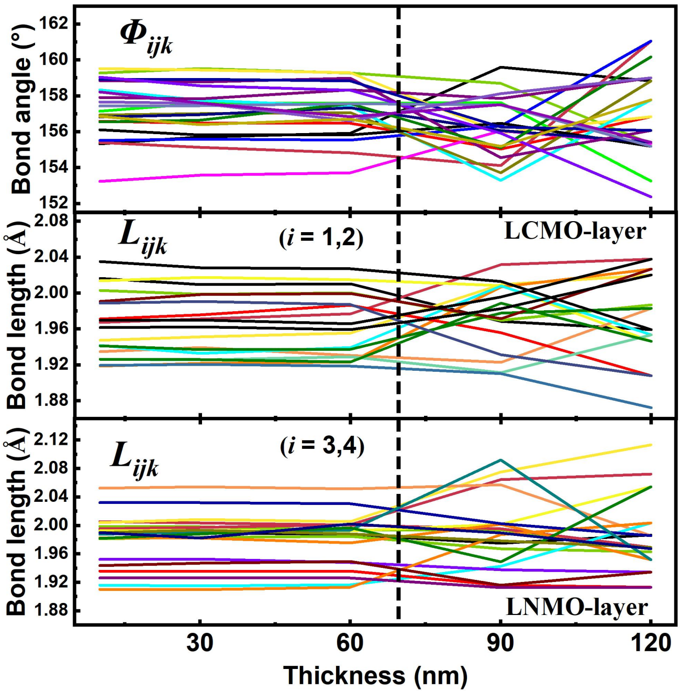

Supplementary Figure S32. The calculated bond lengths (L_{ijk}) and bond angles (Φ_{ijk}) of the SL_{10}, SL_{30}, SL_{60}, SL_{90} and SL_{120} films with different epitaxial strains. The curves of bond length and bond angle converge around 66 nm thickness. In this system, the changes in bond length and bond angle correspond to the tilting and rotation of the oxygen octahedron. Therefore, the tilting and rotation of BO_6 octahedron have an optimal coupling state under the epitaxial strain, obtaining a high structural stability to reduce the extreme effect of bond length or bond angle.

Supplementary Table S2. The calculated bond length (L_{ijk}) of SL films with different thicknesses.

Bond length (Å)	SL-10nm	SL-30nm	SL-60nm	SL-90nm	SL-120nm
L111	2.01648	2.00947	2.01055	1.96851	1.95931
L112	2.00316	1.99868	2.00045	1.96954	1.98717
L113	2.05093	2.05324	2.05384	2.00541	2.00758
L114	2.06547	2.06714	2.06928	2.03657	2.00654
L115	1.96757	1.97134	1.97688	2.03191	2.03782
L116	1.94767	1.95148	1.9557	2.00956	2.02026
L131	2.01376	2.01768	2.01513	2.00833	1.94634
L132	1.91884	1.92182	1.9232	2.00706	2.027
L133	1.93501	1.93927	1.93045	1.92286	1.98318
L134	1.94187	1.93274	1.93959	2.00801	1.95361
L135	1.9712	1.97568	1.9865	1.95579	1.9079

L136	1.90949	1.9113	1.9096	1.87726	1.87246
L211	1.92625	1.92554	1.92922	1.91135	1.95361
L212	1.94126	1.93731	1.93754	1.97775	1.98318
L213	1.99092	1.99865	1.99947	1.97182	2.027
L214	1.92628	1.9259	1.92331	1.98879	1.94634
L215	1.91948	1.9205	1.91877	1.91022	1.87247
L216	1.98874	1.99079	1.9874	1.93142	1.9079
L231	2.06993	2.0726	2.07039	2.08075	2.00654
L232	2.07696	2.07778	2.07496	2.07429	2.00758
L233	2.05588	2.05213	2.05439	2.00693	1.98717
L234	2.03533	2.02841	2.02715	2.01301	1.95931
L235	1.96183	1.96262	1.95943	1.98283	2.02026
L236	1.9696	1.9702	1.96576	1.99579	2.03782
L311	1.99445	1.98999	1.9874	1.97505	1.98674
L312	1.98152	1.98328	1.98506	1.96756	1.96287
L313	1.99907	1.99817	1.99367	1.99409	1.9674
L314	2.0058	2.00378	2.00068	1.99566	1.96927
L315	1.99526	1.99752	2.00136	2.06445	2.07251
L316	2.00433	2.00838	2.00561	2.0752	2.11343
L331	1.99272	1.99355	1.99269	2.00171	2.0544
L332	1.98275	1.98263	1.97542	2.00197	1.95214
L333	2.05266	2.05465	2.05147	2.05739	1.98593
L334	1.91595	1.91537	1.91688	1.94285	2.00351
L335	1.93597	1.9353	1.93577	1.9161	1.9133
L336	1.9524	1.95291	1.94812	1.93799	1.93439
L411	1.91019	1.9097	1.91314	1.98739	2.00351
L412	2.03214	2.03221	2.03064	2.00248	1.98593
L413	1.98757	1.99105	1.9936	2.09205	1.95214
L414	1.98169	1.98813	1.99677	1.94907	2.0544
L415	1.94357	1.94693	1.94942	1.91612	1.93439
L416	1.92633	1.92687	1.92644	1.91258	1.9133
L431	1.99027	1.99355	1.9883	1.97872	1.96927
L432	1.99027	1.98263	2.00203	1.98972	1.9674
L433	1.98649	2.05465	1.98583	1.96831	1.96287
L434	1.99635	1.91537	1.98786	1.95998	1.98674
L435	2.00562	1.9353	2.02202	2.10612	2.11343
L436	1.98659	1.95291	1.99383	2.05299	2.07251

Supplementary Table S3. The calculated bond angle (*Φ_{ijk}*) of SL films with different thicknesses.

Bond angle (°)	SL-10nm	SL-30nm	SL-60nm	SL-90nm	SL-120nm
Φ111	155.3267	155.7141	155.9214	159.5963	158.8367
Φ112	159.281	159.5319	159.2698	158.6937	155.3434
Φ113	156.5901	156.4792	156.4745	155.0383	156.8522
Φ121	155.4041	155.1386	154.8336	154.1136	161.0638
Φ122	157.1567	157.5947	157.5997	157.6333	153.2496
Φ133	159.5235	159.449	159.3005	155.9358	156.8522
Φ211	155.5263	155.5988	155.5418	156.3286	161.0637
Φ212	151.3492	151.41	151.4972	150.7845	153.2496
Φ213	158.3439	157.7539	157.4234	153.2901	157.7823
Φ221	153.239	153.5769	153.7269	156.0646	155.3434
Φ222	156.9368	157.0223	157.0711	153.7217	158.8367
Φ233	156.1146	155.8204	155.8363	156.4825	155.1907
Φ311	156.8327	156.9541	157.3348	156.0672	155.4233
Φ312	157.9206	157.847	158.3429	157.8615	159.0119
Φ313	158.8539	158.7752	158.9897	154.5578	156.0798
Φ321	156.5515	156.6444	157.5803	155.1126	160.1691
Φ322	158.8493	159.2445	160.1538	159.6604	152.3721
Φ333	158.9222	158.9229	158.8293	156.275	156.0798
Φ411	159.0387	158.574	158.3258	155.9133	152.3721
Φ412	163.1255	163.142	162.7849	158.1854	160.1691
Φ413	157.4351	157.4538	157.5739	157.4881	155.1907
Φ421	157.6673	157.5849	156.6132	158.1251	159.0119
Φ422	158.2201	157.6048	156.8388	157.5072	155.4233
Φ433	156.866	156.4047	156.7004	155.2002	157.7823

Supplementary Appendix 1: Landau-Ginsburg-Devonshire theory for ferroelectric phase transition.

The thermodynamic description of strain-induced ferroelectric phase transition in epitaxial thin films is developed from the power-series expansion of the Helmholtz free-energy density F in terms of polarization components P_i and order parameter components q_i[19]. In this system, the order-parameter components q_i were considered as the deviation of positive and negative electric centers caused by the rotation/tilting of BO_6 octahedron. Since the surface polarity of the substrate has a downward guidance on the spontaneous polarization of the films, only z component was retained to simplify the analysis, i.e., $q_1 = q_2 = 0$, $q_3 \neq 0$. The simplified free-energy density F can be expressed as follows[19]:

$$F = \alpha_1(P^2{}_1 + P^2{}_2 + P^2{}_3) + \alpha_{11}(P^4{}_1 + P^4{}_2 + P^4{}_3) + \alpha_{12}(P^2{}_1P^2{}_2 + P^2{}_1P^2{}_3 + P^2{}_2P^2{}_3)$$
$$+ (S^2{}_1 + S^2{}_2 + S^2{}_3)/2c_{11} + c_{12}(S_1S_2 + S_1S_3 + S_2S_3) + (S^2{}_4 + S^2{}_5 + S^2{}_6)/2c_{44}$$
$$- g_{11}(S_1P^2{}_1 + S_2P^2{}_2 + S_3P^2{}_3) - g_{12}[S_1(P^2{}_2 + P^2{}_3) + S_2(P^2{}_1 + P^2{}_3) + S_3(P^2{}_1 + P^2{}_2)]$$
$$- g_{44}(S_4P_2P_3 + S_5P_1P_3 + S_6P_1P_2) - (\lambda_{12}S_1 + \lambda_{12}S_2 + \lambda_{11}S_3)q^2{}_3$$
$$- (t_{12}P^2{}_1 + t_{12}P^2{}_2 + t_{11}P^2{}_3)q^2{}_3 + \beta_1q^2{}_3 + \beta_{11}q^4{}_3\,, \qquad (14)$$

where S_n (n = 1, 2,..., 6) are lattice strains, c_{nl} are the elastic stiffnesses, g_{nl} are the electrostrictive constants, λ_{nl} are the linear-quadratic coupling coefficients between the strain and structural order parameters, and t_{nl} are the coupling coefficients between the polarization and structural order parameters.

For the case of epitaxial strain, the stress comes from the mismatch between the substrate and the film, i.e., $\sigma_3 = 0$, and $S_4 = S_5 = S_6 = 0$. According to the epitaxial mode of $[001]_{DPs}//[001]_{STO}$, only b-axis mismatch of superlattices La_2CoMnO_6/La_2NiMnO_6 is less than 1‰, which approximates $S_2 = 0$, while $S_1 \neq 0$, $S_3 \neq 0$. Corresponding to q_i, the z component of polarization was considered, i.e., $P_1 = P_2 = 0$, $P_3 \neq 0$. Therefore, the Eq. (14) can be further written as:

$$F = [\alpha_1 - (g_{11}S_3 + g_{12}S_1) - t_{11}q^2{}_3]P^2{}_3 + \alpha_{11}P^4{}_3 + c_{11}(S^2{}_1 + S^2{}_3)/2$$
$$+ c_{12}S_1S_3 - (\lambda_{11}S_3 + \lambda_{12}S_1 - \beta_1)q^2{}_3 + \beta_{11}q^4{}_3. \qquad (15)$$

According to the mechanical boundary condition of $\partial F/\partial S_3 = 0$, we then have the following relations:

$$S_1 = (g_{11}P^2{}_3 + \lambda_{11}q^2{}_3 - c_{11}S_3)/c_{12}\,. \qquad (16)$$

Using the relation (16) in Eq. (15), we can simplify the formula of free energy

density with renormalized expansion coefficients, as the following format:

$$F = \alpha^*_{11} P^4_3 + \alpha^*_1 P^2_3 + K, \tag{17}$$

where $\alpha^*_{11} = \alpha_{11} - g_{11}(g_{12} - c_{11}g_{11}/2c_{12})/c_{12}$, $\alpha^*_1 = \alpha_1 + [g_{12}(c_{11}/c_{12}) - g_{11}(c_{11}/c_{12})^2]S_3 - \gamma_1 q^2_3$, $K = [c_{11}(c_{11} + c_{12})(c_{11} - c_{12})/2c^2_{12}]S^2_3 + \gamma_2 q^2_3 + \gamma_3 q^4_3$; $\gamma_1 = t_{11} + (\lambda_{11}g_{12} + \lambda_{12}g_{11})/c_{12} - \lambda_{11}g_{11}c_{11}/c^2_{12}$, $\gamma_2 = \beta_1 + \lambda_{12}S_3(c_{11}/c_{12}) - \lambda_{11}S_3(c_{11}/c_{12})^2$, $\gamma_3 = \beta_{11} + c_{11}(\lambda_{11}/c_{12})^2/2 - \lambda_{11}\lambda_{12}/c_{12}$; $g_{11} = c_{11}Q_{11} + 2c_{12}Q_{12}$, $g_{12} = c_{11}Q_{12} + c_{12}(Q_{11} + Q_{12})$, $Q_{11} = k_1 Q_{eff}$, $Q_{12} = k_2 Q_{eff}$. Here, Q_{nl} are components of electrostrictive constants, Q_{eff} is the estimated value from the experiment, and q_3 is estimated from the statistical average displacement of positive and negative ions within the strained crystal structure calculated by DFT.

In addition, since such improper ferroelectricity in superlattices was dependent on epitaxial strain and guided by the polar surface of the substrate, the energy change for the electric field induced by the dipole moments should be considered. The relevant expressions were as follows[20, 21]:

$$E_d = -2\lambda_e P/\varepsilon_0 d, \tag{18}$$

$$f_{elec} = -\, g_t E_d P/2, \tag{19}$$

where E_d and f_{elec} is the electric field and electric energy density for the evolution of the dipole moments, respectively; λ_e is the effective thickness of the surface capacitance layer, and d is the film thickness. The effective influence factor g_t is related to the polarizability of the materials, and given by $k_3/(\varepsilon_r-1)$.

For simultaneous equations of Eqs. (17), (18) and (19), the total energy density F^* was rewritten as:

$$F^* = \alpha^*_{11} P^4_3 + [\alpha^*_1 + k_3\lambda_e/(\varepsilon_r - 1)\varepsilon_0 d]P^2_3 + K. \tag{20}$$

According to the spontaneous polarization $P_s = P_3 |_{\partial F^*/\partial P = 0}$, we could have the value c_{11}=4.535529018×10^9 N/m^2, c_{12}=6.258271904×10^9 N/m^2, γ_1=-2.464744762×10^9 1/Fm, when the following coefficients were adopted: α_1=1.92894×10^4 m/F, α_{11}=5.0×10^{10} m^5/C^{2}F, λ_e = 0.2 Å, Q_{eff}=4.97×10^{-3} m^4/C^2, k_1=1.0, k_2=-0.3, k_3=2.0005, ε_0=8.8541878×10^{-12} F/m, $q_{3\text{-}30}$=0.3149 Å, $q_{3\text{-}60}$=0.3148 Å, and $q_{3\text{-}90}$=0.3138 Å. Here, the coefficient $\alpha_1 = \alpha(T)|_{T=300\text{K}}$ since the P-E loops were measured at room temperature. γ_1 was solved as a whole coefficient, rather than a polynomial. The curves of $\Delta E(P)$-P with ferroelectric phase transitions were plotted in Figure 4f.

Supplementary References

1. S. R. Provence, et al. Machine learning analysis of perovskite oxides grown by molecular beam epitaxy. *Phys. Rev. Mater.* **4**, 083807 (2020).
2. M. Hashisaka, et al. Epitaxial growth of ferromagnetic La_2NiMnO_6 with ordered double-perovskite structure. *Appl. Phys. Lett.* **89**, 032504 (2006).
3. H. S. Wu, et al. Metal-free perovskite piezoelectric nanogenerators for human-machine interfaces and self-powered electrical stimulation applications. *Adv. Sci.* **9**, 2105974 (2022).
4. J. Xiao, W. L. Ong, Z. Guo, G. W. Ho & K. Zeng. Resistive switching and polarization reversal of hydrothermal-method-grown undoped zinc oxide nanorods by using scanning probe microscopy techniques. *ACS Appl. Mater. Interfaces* **7**, 11412-22 (2015).
5. Y. Jiang, et al. Gradient strain-induced room-temperature ferroelectricity in magnetic double-perovskite superlattices. *Small Methods* **7**, 2201246 (2023).
6. Z. Chen, et al. ε-Ga_2O_3 : an emerging wide band gap piezoelectric semiconductor for application in radio frequency resonators. *Adv. Sci.* **9**, e2203927 (2022).
7. Y. Yan, et al. Ultrahigh piezoelectric performance through synergistic compositional and microstructural engineering. *Adv. Sci.* **9**, 2105715 (2022).
8. A. Song, et al. Simultaneous enhancement of piezoelectricity and temperature stability in KNN-based lead-free ceramics via layered distribution of dopants. *Adv. Funct. Mater.* **32**, 2204385 (2022).
9. Y. C. Yang, C. Song, X. H. Wang, F. Zeng & F. Pan. Giant piezoelectric d_{33} coefficient in ferroelectric vanadium doped ZnO films. *Appl. Phys. Lett.* **92**, 012907 (2008).
10. Q. Shi, et al. The role of lattice dynamics in ferroelectric switching. *Nat. Commun.* **13**, 1110 (2022).
11. M. A. Magray, M. Ikram & M. Najim. Impact of oxygen vacancies to control the magnetic and electronic properties of the La_2CoMnO_6 system. *J. Magn. Magn. Mater.* **529**, 167857 (2021).
12. M. Jiang, et al. Double perovskites as model bifunctional catalysts toward rational design: the correlation between electrocatalytic activity and complex spin configuration. *ACS Appl. Mater. Interfaces* **10**, 19746-19754 (2018).
13. Y. Tong, et al. Vibronic superexchange in double perovskite electrocatalyst for

efficient electrocatalytic oxygen evolution. *J. Am. Chem. Soc.* **140**, 11165-11169 (2018).

14. H. Z. Guo, et al. Influence of defects on structural and magnetic properties of multifunctional La_2NiMnO_6 thin films. *Phys. Rev. B* **77**, 174423 (2008).
15. P. R. Mandal, A. Khan & T. K. Nath. Antisite disorder driven magnetodielectric and magnetocaloric effect in double perovskite $La_{2-x}Sr_xCoMnO_6$ ($x = 0.0$, 0.5, 1.0). *J. Appl. Phys.* **128**, 024104 (2020).
16. H. Gan, C. Wang & Q. Shen. Improved magnetic performance of Co-doped La_2NiMnO_6 ceramics prepared at low temperature. *J. Eur. Ceram. Soc.* **40**, 1909-1916 (2020).
17. C. Meyer, V. Roddatis, P. Ksoll, B. Damaschke & V. Moshnyaga. Structure, magnetism, and spin-phonon coupling in heteroepitaxial $La_2CoMnO_6/Al_2O_3(0001)$ films. *Phys. Rev. B* **98**, 134433 (2018).
18. Y. Zhang, M. P. K. Sahoo, T. Shimada, T. Kitamura & J. Wang. Strain-induced improper ferroelectricity in Ruddlesden-Popper perovskite halides. *Phys. Rev. B* **96**, 144110 (2017).
19. R. Xu, et al. Strain-induced room-temperature ferroelectricity in $SrTiO_3$ membranes. *Nat. Commun.* **11**, 3141 (2020).
20. G. Gerra, A. K. Tagantsev, N. Setter & K. Parlinski. Ionic polarizability of conductive metal oxides and critical thickness for ferroelectricity in $BaTiO_3$. *Phys. Rev. Lett.* **96**, 107603 (2006).
21. Zibin Chen1, et al. Giant tuning of ferroelectricity in single crystals by thickness engineering. *Sci. Adv.* **6**, eabc7156 (2020).
