# Supplementary material for: Experimental demonstration of tunable hybrid improper ferroelectricity in double-perovskite superlattice films
Source: Nat Commun. 2024 Jul 2;15:5549. doi: 10.1038/s41467-024-49707-x (PMC11219787; doi:10.1038/s41467-024-49707-x)
Supplement: Supplementary file 3 — Reporting Summary [file 41467_2024_49707_MOESM3_ESM.pdf]

nature research

Corresponding author(s): Weibo Gao

Lasing Reporting Summary

Nature Research wishes to improve the reproducibility of the work that we publish. This form is intended for publication with all accepted papers reporting claims of lasing and provides structure for consistency and transparency in reporting. Some list items might not apply to an individual manuscript, but all fields must be completed for clarity.

For further information on Nature Research policies, including our data availability policy, see Authors & Referees.

• Experimental design

Please check: are the following details reported in the manuscript?

1. Threshold

Item	Response	Details
Plots of device output power versus pump power over a wide range of values indicating a clear threshold	☐ Yes	State where this information can be found in the text.
	☒ No	Explain why this information is not reported/not relevant.

2. Linewidth narrowing

Item	Response	Details
Plots of spectral power density for the emission at pump powers below, around, and above the lasing threshold, indicating a clear linewidth narrowing at threshold	☐ Yes	State where this information can be found in the text.
	☒ No	Explain why this information is not reported/not relevant.
Resolution of the spectrometer used to make spectral measurements	☐ Yes	State where this information can be found in the text.
	☒ No	Explain why this information is not reported/not relevant.

3. Coherent emission

Item	Response	Details
Measurements of the coherence and/or polarization of the emission	☐ Yes	State where this information can be found in the text.
	☒ No	Explain why this information is not reported/not relevant.

4. Beam spatial profile

Item	Response	Details
Image and/or measurement of the spatial shape and profile of the emission, showing a well-defined beam above threshold	☐ Yes	State where this information can be found in the text.
	☒ No	Explain why this information is not reported/not relevant.

5. Operating conditions

Item	Response	Details
Description of the laser and pumping conditions *Continuous-wave, pulsed, temperature of operation*	☒ Yes	248nm, 2Hz, room temperature
	☐ No	Explain why this information is not reported/not relevant.
Threshold values provided as density values (e.g. W cm^{-2} or J cm^{-2}) taking into account the area of the device	☒ Yes	2 J/cm2
	☐ No	Explain why this information is not reported/not relevant.

6. Alternative explanations

Item	Response	Details
Reasoning as to why alternative explanations have been ruled out as responsible for the emission characteristics *e.g. amplified spontaneous, directional scattering; modification of fluorescence spectrum by the cavity*	☐ Yes	State where this information can be found in the text.
	☒ No	Explain why this information is not reported/not relevant.

7. Theoretical analysis

Item	Response	Details
Theoretical analysis that ensures that the experimental values measured are realistic and reasonable *e.g. laser threshold, linewidth, cavity gain-loss, efficiency*	☐ Yes	State where this information can be found in the text.
	☒ No	Explain why this information is not reported/not relevant.

8. Statistics

Item	Response	Details
Number of devices fabricated and tested	☐ Yes	State where this information can be found in the text.
	☒ No	Explain why this information is not reported/not relevant.
Statistical analysis of the device performance and lifetime (time to failure)	☐ Yes	State where this information can be found in the text.
	☒ No	Explain why this information is not reported/not relevant.
